# Advertising of orthodontic appliances on websites in the UK: Do they comply with advertising standards? A cross-sectional study

**DOI:** 10.1177/14653125251408302

**Published:** 2026-02-26

**Authors:** Arunika Nehra, Adam Jones, Trevor Hodge

**Affiliations:** 1 General Dental Practitioner, NHS, London, UK; 2 Department of Oral Surgery, University of Leeds, Leeds, UK; 3 Department of Orthodontics, Leeds Teaching Hospitals NHS Trust, Leeds, UK

**Keywords:** orthodontics, aligners, direct-to-consumer, tele-dentistry, advertising, quality of information

## Abstract

**Aim:**

To evaluate the compliance of websites promoting proprietary orthodontic appliances available in the UK against advertising standards outlined by the Advertising Standards Authority Committee of Advertising Practice (ASA CAP) Code.

**Design:**

Cross-sectional study.

**Setting:**

Websites promoting proprietary orthodontic appliances available in the UK, including fixed, removable and aligner systems sold as a complete system under a brand name.

**Methods:**

A comprehensive, systematic approach was adopted, beginning with a 2020 scoping search on Google and social media platforms (Instagram and Facebook) to identify keywords. Keyword searches were conducted on Google in 2020, 2023 and 2025 to identify relevant websites. To ensure contemporary relevance, only websites identified in the final 2025 search were included for analysis. Raters underwent training and calibration before independently evaluating websites for compliance with advertising standards using bespoke judgement criteria derived from the ASA CAP Code, across four domains: comprehensiveness of treatment information; presentation of treatment information; objectivity of treatment information; and substantiation of claims. Discrepancies were resolved through group discussion to determine agreed scores. Data were analysed using descriptive and inferential statistics (Fleiss’ kappa and Kruskal–Wallis tests).

**Results:**

The 2025 search identified 970 websites, of which 39 met the inclusion criteria. Inter-rater reliability showed almost perfect agreement (kappa >0.9). Compliance varied significantly across domains: 45% of all claims provided comprehensive information, 54% had clear presentation, 38% maintained objectivity and only 4% of claims were substantiated with evidence. Nearly all websites (95%) omitted common risks and 92% failed to mention alternative treatments. Direct-to-consumer and tele-dentistry websites showed poorer compliance than dentist-delivered systems.

**Conclusions:**

Orthodontic appliance websites showed poor compliance with ASA CAP Code standards. The majority used descriptive language and words in place of numbers to quantify magnitude, alongside subjective content and unsubstantiated claims, with omissions of treatment risks. These findings raise significant concerns about online orthodontic advertising and its potential impact on informed patient decision-making.

## Plain language summary

How well do UK websites for orthodontic appliances follow advertising rules?

**Why was the study done?** Orthodontic appliances, like braces and aligners, are increasingly marketed online in the UK, but it is unclear whether these websites follow advertising rules. The Advertising Standards Authority (ASA) has set guidelines to ensure ads are honest and clear. This study looked at whether orthodontic websites are meeting these standards to protect consumers. **What did the researchers do?** The research team examined 39 UK-based orthodontic websites that promote braces and aligners. They checked these websites for compliance with the ASA’s advertising rules, which focus on the treatment information provided to be clear, accurate, and evidence based. They also assessed whether important information about common treatment risks, side effects, contraindications and alternate treatments was included. **What did the researchers find?** The study revealed several issues with the advertising on orthodontic appliance websites. Overall, only 45% of websites provided comprehensive treatment information. More than half (54%) presented clear information, but many used vague language like ‘fast’ or ‘painless’ without clarity. All websites (39/39) included patient and/or dentist testimonials to promote the appliance, and only 4% of claims were backed by evidence. Websites for dentist-delivered aligners performed better in meeting standards and were the only appliance to include some (9%) substantiated claims. Direct-to-consumer aligners and tele-dentistry sites had more unsubstantiated claims and missing information. **What do the findings mean?** This study highlights the need for better advertising practices by websites of orthodontic appliances. It shows that many websites fail to provide reliable and evidence-based information, which could mislead consumers. Improved transparency and adherence to advertising standards are necessary to help people make informed choices about orthodontic treatments.

## Introduction

The direct-to-consumer (DTC) model of orthodontic treatment, which does not require an in-person examination by an orthodontist, is becoming more prevalent in the United Kingdom (UK) (British Orthodontic Society, 2014; Kravitz and Bowman, 2016; Rahman et al., 2020; Wexler et al., 2020). The degree of professional oversight varies significantly, with some providers offering tele-dentistry supervision (British Orthodontic Society, 2022) and others adopting a ‘do-it-yourself’ approach (Carter and Stokes, 2021). Treatment objectives also differ, ranging from comprehensive treatment aimed at correcting all aspects of malocclusion to those with ‘limited objectives’, such as aligning the upper anterior teeth only (McDonald, 2017; Noar et al., 2015).

Accurate information is critical when consumers are faced with making healthcare decisions with limited or no access to professional guidance. However, a growing body of evidence suggests that the quality of information from orthodontic appliance providers often falls short of accepted standards (Alkadhimi et al., 2022; Alpaydin et al., 2021; Alsaqabi et al., 2023; Hameed et al., 2021; Meade et al., 2022, 2023, 2024).

Several studies have applied frameworks such as DISCERN (Charnock et al., 1999) and LIDA (Coulter et al., 2008) to websites offering orthodontic appliances, and have frequently reported poor reliability, quality and readability of orthodontic website information (Meade and Dreyer, 2022; Meade et al., 2022, 2023, 2024; Parekh and Gill, 2014; Patel and Cobourne, 2015). Concerns persist within the orthodontic community regarding misleading claims on orthodontic websites that do not meet General Dental Council (GDC) standards, potentially creating unrealistic patient expectations (Alsaqabi et al., 2023; Parekh and Gill, 2014; Patel and Cobourne, 2015; Stanford, 2017). Assessments of DTC aligner companies have revealed that consent based solely on website information is likely invalid (Meade and Dreyer, 2021) and claims about the treatment are often unsupported by evidence (Alkadhimi et al., 2022; Meade et al., 2024).

A recent study by Meade et al. (2024) evaluated the quality and accuracy of information presented on websites of marketed orthodontic product providers and found that only 8% of statements made were objectively true. One UK study reported that only 11% of orthodontic aligner websites fully complied with GDC guidelines for ethical advertising (Alsaqabi et al., 2023). Internationally, orthodontic practice websites have similarly shown poor compliance with legal and regulatory standards (Meade et al., 2023).

The Advertising Standards Authority (ASA) is the UK advertising regulator and enforces the Committee of Advertising Practice (CAP) Code rules. The CAP Code (2019) is the UK rulebook for non-broadcast advertisements. It states that, ‘*marketing communications must not mislead or exaggerate, omit material information or foster ambiguity, present subjective claims as objective, or make unsubstantiated claims*’. Although the ASA lacks direct statutory enforcement powers, it employs a range of escalating sanctions, including requiring the removal or amendment of online content, restricting advertising placements and referring serious breaches to statutory bodies such as National Trading Standards. The publication of rulings and sanctions further promotes compliance by impacting advertisers’ reputations (CAP Code, 2019). As of May 2025, the ASA has investigated three orthodontic aligner websites accused of making unsubstantiated claims about orthodontic treatment, in each case ruling that there was insufficient evidence to support the claims investigated (Advertising Standards Authority, 2018). Several more recent cases have been informally resolved (Advertising Standards Authority, 2025). A comprehensive assessment of orthodontic appliance websites’ compliance with the ASA guidelines has yet to be undertaken.

The CAP Code has been applied in studies to evaluate the compliance of websites with advertising standards (Donnell et al., 2021; Stead et al., 2021). To date, no study has comprehensively evaluated orthodontic provider websites against advertising standards. Unlike previous studies that have used tools such as DISCERN and LIDA to assess website quality, readability and the reliability of health information (Meade and Dreyer, 2022; Parekh and Gill, 2014; Patel and Cobourne, 2015), the CAP Code incorporates specific healthcare advertising provisions to ensure regulatory compliance and safeguard consumers against misleading advertising.

This study aimed to evaluate websites of orthodontic appliances available in the UK from a consumer perspective, using an objective framework based on the CAP Code. The term ‘orthodontic appliance’ has been used to denote all proprietary orthodontic appliances, including fixed, removable and aligner systems that are sold as a complete system under a brand name.

The objectives were to identify potential shortcomings in the comprehensiveness and presentation of orthodontic treatment information, assess the objectivity of information and evaluate the substantiation of claims made.

## Materials and methods

This was a cross-sectional study. Ethical approval was not required for this study as it involved publicly available information from orthodontic appliance websites.

### Search strategy

An initial scoping search of Google and social media platforms (Instagram and Facebook) was conducted from a UK-based computer on 10 July 2020 to identify keywords. Social media searches focused on advertisements within three months of the search date. Keywords ‘Orthodontic Braces’, ‘Orthodontic Aligner’, ‘Orthodontic Appliance’ and ‘Clear Aligner’ were identified. No additional relevant websites were identified through the social media searches, so subsequent searches focused on Google only.

Using the identified keywords, comprehensive Google searches were performed on 10 July 2020, repeated on 20 March 2023 and updated on 10 May 2025 during manuscript preparation to ensure data currency, given the rapid proliferation of orthodontic provider websites. To ensure contemporary relevance, only websites identified in the updated May 2025 search were included for analysis. The search was limited to websites written in the English language and the first 1,000 websites for each keyword. Sponsored advertisements were excluded. See Table 1 for website eligibility.

**Table 1.** Eligibility criteria for websites.

Inclusion	Exclusion
Websites for proprietary orthodontic systems that are available in the UK, including: • Fixed appliances • Removable appliances • Aligners Websites aimed at patients	Websites of individuals, e.g. dentists, orthodontists Websites for dental/orthodontic practices Websites for dental/orthodontic laboratories Websites aimed at dental professionals Websites aimed at orthodontic specialists Opinion articles Blogs Presentations of patient cases

### Selection of eligible websites

Two raters (AN and SKB) independently screened websites for eligibility using predefined criteria (Table 1). Disagreements were resolved through discussion with a third rater (TMH).

### Development of bespoke judgement criteria

The CAP Code (Edition 12) rules served as the foundation for devising a bespoke judgement tool (Table 2) to assess website advertising compliance across four domains:

- Comprehensiveness of treatment information
- Presentation of treatment information
- Objectivity of treatment information
- Substantiation of claims

**Table 2.** Criteria for judging advertising quality of websites.

Judgement criteria		Descriptor and examples	Scoring system
Comprehensiveness of treatment information *Is all expected information included?*	Aim of treatment	The purpose of treatment is stated, e.g., to straighten the teeth, to fully correct the teeth and bite.	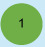 Complete information
	Mode of action	Includes a description of how the appliance works, e.g., moves teeth incrementally through a series of aligners.	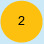 Partial information
	Scope of treatment	Indicates the type of malocclusion that can be treated, e.g., mild crowding.	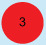 No information
	Contraindications	Indicates any contraindications to treatment, e.g., severe crowding, poor dental health, age restrictions.	
	Alternative treatments	Explicitly states that other types of appliances or approaches may be a reasonable alternative treatment, e.g., there may be other braces that are also suitable to straighten your teeth.	
	Requirements for success	Describes behaviours that are necessary for success, e.g., hours of wear, change in diet, regular checks.	
	Likely treatment time	Provides an estimate of average and range of treatment time.	
	Need for long-term retention	Explicitly states long-term orthodontic retention required to maintain tooth position.	
	Common risks of treatment	Highlights common risks of treatment, e.g., root resorption, decalcification, periodontal damage.	
	Common side effects of treatment	Highlights common side effects of treatment, e.g., discomfort, impact on speech.	
Presentation of treatment information *Is information clear, accessible and consistent?*	Accessibility of information	Information is available and easy to find and does not require navigation to another website.	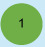 Yes
			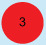 No
	Consistency of information	Information in FAQs is consistent with information on the main website.	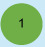 Yes 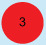 No 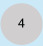 No FAQ
	Clarity of information	Does not use descriptive language that is open to interpretation and could therefore be misleading. Examples include: faster results, effective treatment, painless. Does not use words in the place of numbers to quantify magnitude. Examples include: far more efficient than traditional braces, most advanced aligner.	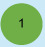 Yes 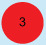 No
Objectivity of treatment information *Are the claims that consumers are likely to regard as objective, able to be objectively substantiated?*	Use of objective information	Where subjective information is used (such as patient or dentist testimonials), this information is: • Proportionate to objective information • Clearly signposted/labelled Allows unedited user reviews.	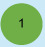 Yes 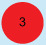 No
	Use of professional photographs	Photographs are of a high quality. Photographs are representative: teeth are shown in occlusion and reasonable range of cases and outcomes shown. Photographs allow a fair before-and-after comparison, and any adjunctive treatment has been stated.	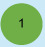 Yes 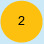 Partial 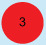 No 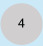 Not used
	Use of patient photographs	Photographs are of a reasonable quality.	
Substantiation of claims *Are there unsubstantiated claims or exaggerations about the capability or performance of a product?*	Materials or design	Claims about materials or design, e.g., superior technical performance.	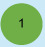 Claims are supported by appropriate evidence
	Treatment process	Claims about treatment process, e.g., discomfort, speech, impact on lifestyle.	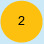 Claims are reasonable but not supported by evidence
	Outcome	Claims about treatment outcome, e.g., improved confidence, improved dental health.	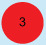 Claims are exaggerated or use inappropriate citation
	Superiority to comparators	Claims made about treatment or appliance compared to alternative treatments or appliances.	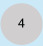 No claims are made

FAQ, frequently asked question.

### Piloting and calibration of judgement criteria

The development process for the judgement criteria involved all raters (AN, SKB, TMH and AJ) first agreeing on definitions for each criterion, then piloting and iteratively modifying the criteria.

### Training and calibration of raters

All raters underwent training and calibration by comparing and discussing the scores made by two raters (AN and AJ) independently for a sample of websites. Any disagreements were discussed and resolved by two independent raters (SKB and TMH). The calibration process devised descriptors and examples for each judgement criterion to ensure scoring consistency.

### Data extraction

Data were independently extracted by two raters (AN and SKB), entered into Microsoft Excel (Version 16.69.1, UK) and then verified by a third rater (TMH).

### Data analysis

Websites were grouped based on the category of orthodontic appliance (Table 3). All raters independently scored each website against the judgement criteria, assigning a score from 1 to 4, as outlined in Table 2. Any disagreements were resolved through group discussion. A summary of individual rater scores is provided in the supplemental material.

**Table 3.** Categorisation of orthodontic appliances.

Category	Description
Dentist-delivered aligners	Aligner treatment that is delivered by a dentist and involves in-person visits.
Fixed appliances	Fixed appliance system that is sold as a complete system of brackets and wires.
Multiple appliance systems	Companies that offer different appliance systems, for example, fixed and aligner systems, within the same branding.
Tele-dentistry	Treatment that is planned and delivered remotely, for example, by the patient sending photographs or videos.
Direct-to-consumer	Treatment that does not involve input from a dental professional. This includes systems where scans are taken in-store but without a dental consultation.

**Figure 1.**
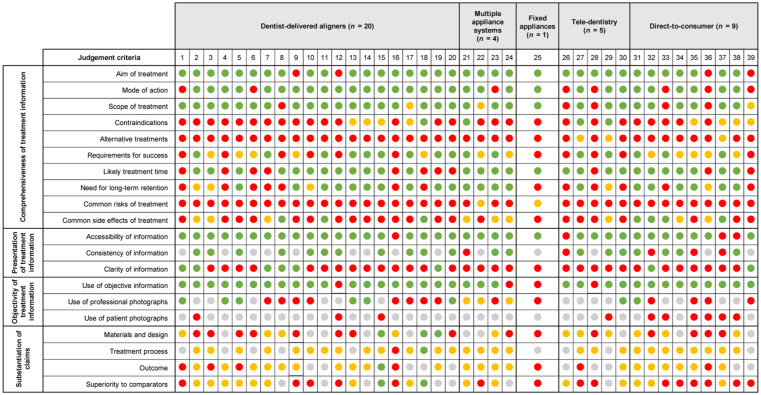
Summary of final consensus scores for all websites. 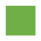
 Complete information/yes/claims are supported by appropriate evidence. 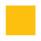
 Partial information/partial/claims are reasonable but not supported by evidence. 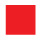
 No information/no/claims are exaggerated or use inappropriate citation. 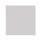
 No FAQ/not used/no claims are made.

A final consensus score was then agreed by the four raters for each judgement sub-criterion through group discussion (see Figure 1).

### Statistical analysis

The data were analysed using Microsoft Excel (Version 16.69.1, UK) and R statistical software (Version 4.3.1, R Core Team, 2023).

Fleiss’ kappa was applied at a 5% significance level to measure inter-rater agreement across the scores (see Table 4). Overall and criterion-specific kappa values, along with their 95% confidence intervals (CIs), were estimated. Fleiss’ kappa was chosen due to the ordinal scores and multiple raters (Fleiss, 1971).

**Table 4.** Fleiss’ kappa values assessing inter-rater agreement across scores.

Judgement criteria	kappa value	95% CI		*p* value
		Lower bound	Upper bound	
Comprehensiveness of treatment information	0.902	0.863	0.942	*p* < 0.001
Presentation of treatment information	0.945	0.891	0.999	*p* < 0.001
Objectivity of treatment information	0.987	0.963	0.999	*p* < 0.001
Substantiation of claims	0.946	0.902	0.989	*p* < 0.001
Overall judgement across all criteria	0.939	0.919	0.960	*p* < 0.001

CI, confidence interval.

Given the ordinal nature of the data, a post-hoc analysis was conducted using the non-parametric Kruskal–Wallis test and Dunn's multiple comparison test (with Bonferroni correction) to compare median scores across the four main judgement criteria and the five orthodontic appliance categories.

## Results

The updated May 2025 search identified 970 websites, of which 39 were assessed as eligible for inclusion: 20 dentist-delivered aligners, four multiple appliance systems, one fixed appliance, five tele-dentistry and nine DTC appliances.

**Figure 2.**
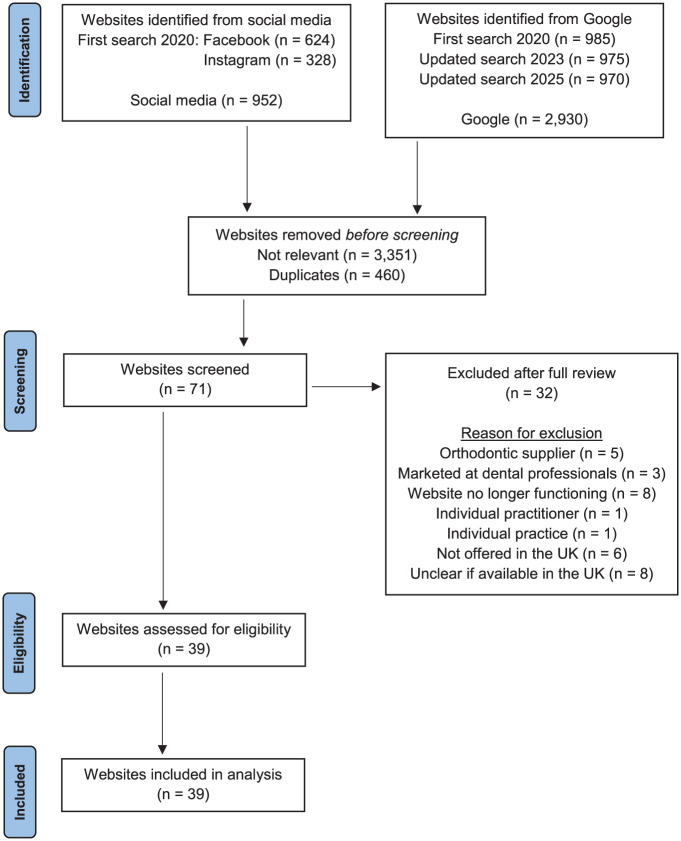
Flowchart of the identification and selection of websites.

See Figure 2 for a flowchart of the methodology.

### Inter-rater reliability and statistical analysis of scores

**Figure 3.**
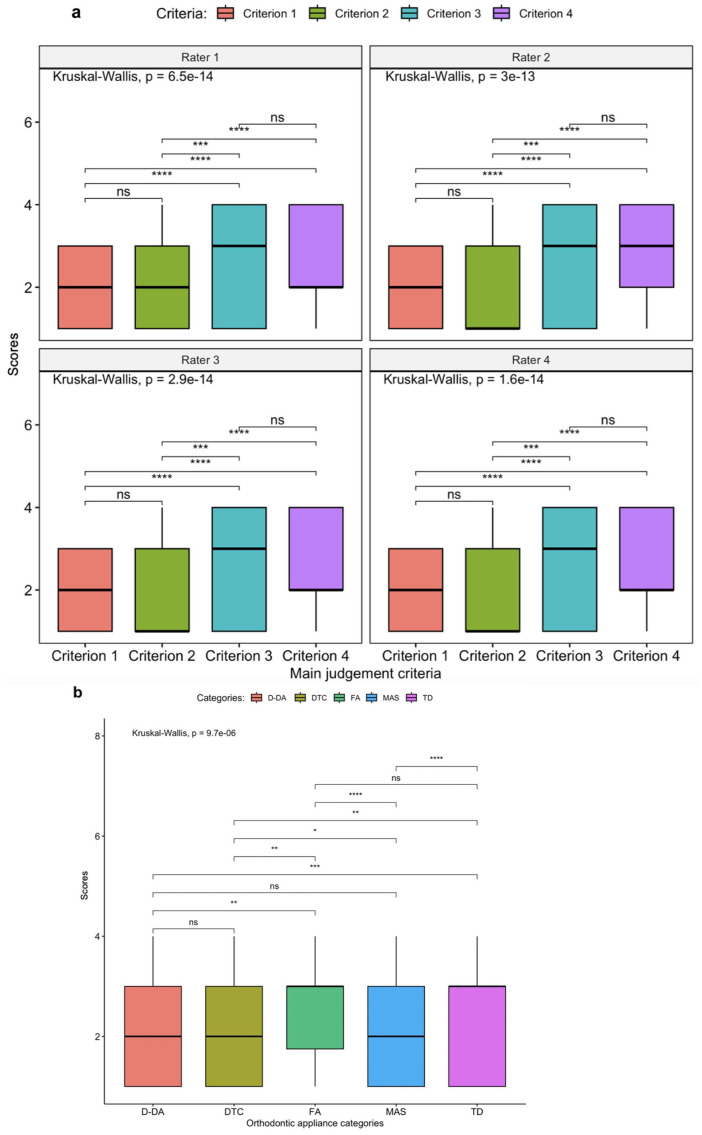
Box plots showing the distribution of scores across (a) the four main judgement criteria and (b) the five orthodontic appliance categories. Significant differences were identified using the Kruskal–Wallis test (*p* < 0.001), followed by Dunn’s multiple comparison test with Bonferroni correction. Statistical significance is annotated on the plots. Criterion 1 = comprehensiveness of treatment information; criterion 2 = presentation of treatment information; criterion 3 = objectivity; criterion 4 = substantiation of claims. *p < 0.05, **p < 0.01, ***p < 0.001 and ****p < 0.0001. Scores: 1 = complete information/yes/claims are supported by appropriate evidence; 2 = partial information/partial/claims are reasonable but not supported by evidence; 3 = no information/no/claims are exaggerated or use inappropriate citation; 4 = no FAQ/not used/no claims are made. D-DA, dentist-delivered aligners; DTC, direct-to-consumer; FA, fixed appliances; FAQ, frequently asked question; MAS, multiple appliance systems; ns, non-significant; TD, tele-dentistry.

Almost perfect agreement was found across the judgement criteria, with a Fleiss’ kappa of 0.939 (95% CI = 0.919–0.960; *p* < 0.001), as shown in Table 4. A post-hoc analysis using the Kruskal–Wallis test indicated significant differences in median scores between the four main judgement criteria for each rater (*p* <0.001) (Figure 3a). In addition, a significant variation in the distribution of scores across the five orthodontic appliance categories was observed (*p* < 0.001) (Figure 3b).

## Summary of treatment information across websites

The results are reported by websites and claims: when summarising findings in terms of websites, both the number (n) and percentage (%) are provided; when reporting the claims within the websites, only the percentage (%) is used.

### Comprehensiveness of treatment information

**Figure 4.**
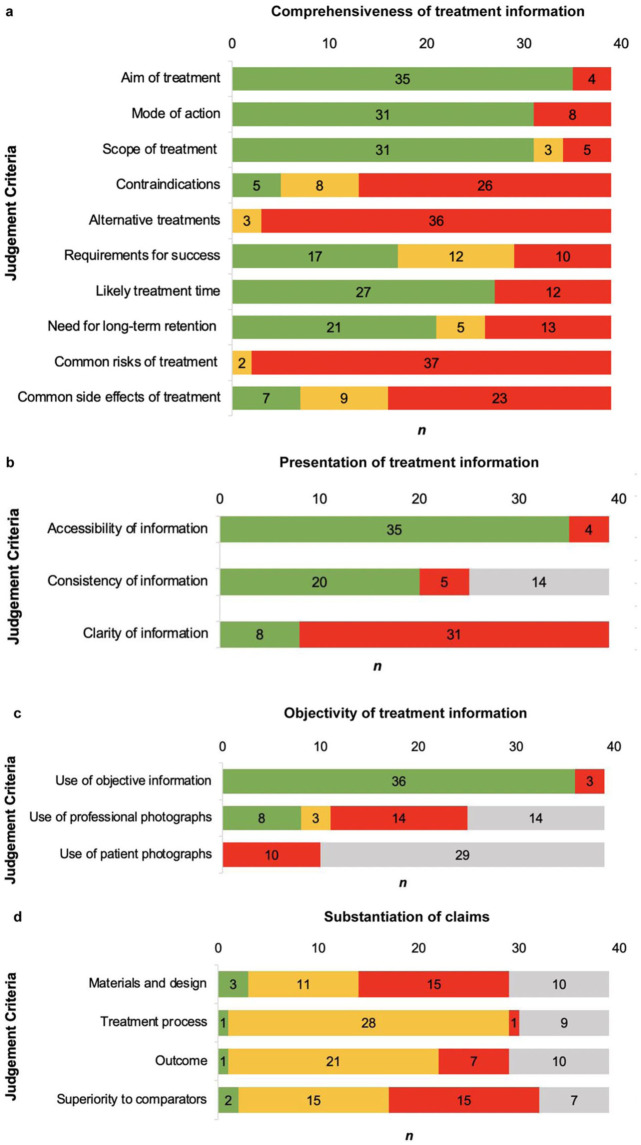
(a–d) Subgraphs illustrating scores across the four main judgement criteria and their corresponding sub-criteria: (a) comprehensiveness of treatment information; (b) presentation of treatment information; (c) objectivity of treatment information; and (d) substantiation of claims. 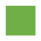
 Complete information/yes/claims are supported by appropriate evidence. 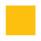
 Partial information/partial/claims are reasonable but not supported by evidence. 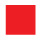
 No information/no/claims are exaggerated or use inappropriate citation. 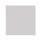
 No FAQ/not used/no claims are made.

**Figure 5.**
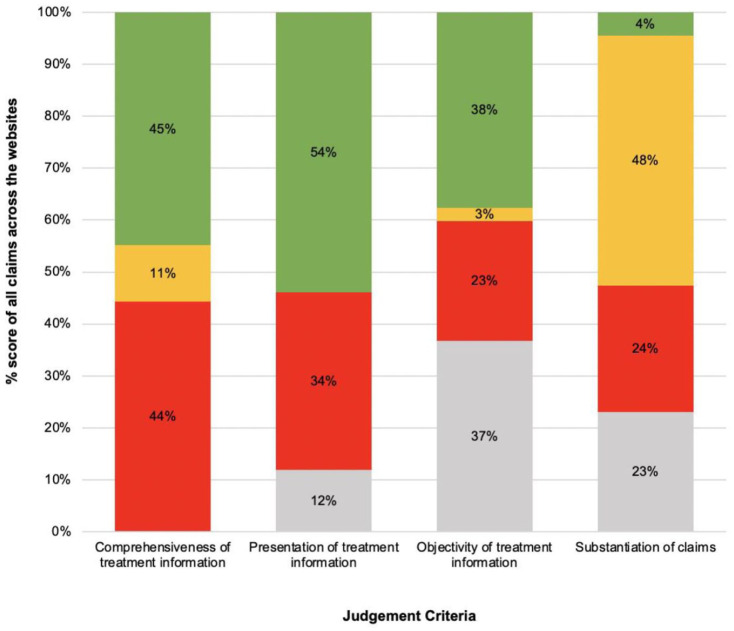
Summary of scores across the four main judgement criteria. Note: All percentages may not total 100% due to rounding. 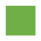
 Complete information/yes/claims are supported by appropriate evidence. 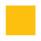
 Partial information/partial/claims are reasonable but not supported by evidence. 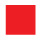
 No information/no/claims are exaggerated or use inappropriate citation. 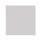
 No FAQ/not used/no claims are made.

Figure 4a summarises the comprehensiveness of treatment information on websites. As shown in Figure 5, nearly half (45%) of the claims made within websites provided comprehensive information about the treatment. Figure 4a shows that the best-reported items on websites were aim (n = 35, 90%), mode (n = 31, 85%), scope of treatment (n = 31, 85%), likely treatment time (n = 27, 72%) and the need for long-term retention (n = 21, 56%). Figure 5 also highlights that 44% of the claims within websites included no information about the treatment. Figure 4a shows that the worst-reported items were common risks (n = 37, 95%), alternative treatments (n = 36, 92%), contraindications (n = 26, 67%) and common side effects (n = 23, 59%). Websites often omitted side effects, such as discomfort and impact on speech.

Some websites (n = 12, 31%) included partial information about the requirements for success but omitted either the need for regular dental checks or the number of hours aligners should be worn. Five websites (13%) included complete information on contraindications, stating that treatment was not recommended in patients with severe periodontal disease, was limited to anterior teeth and severe dental misalignment was not treatable. Eight websites (21%) included partial information, highlighting the need to assess oral hygiene before treatment and stating age restrictions.

### Presentation of treatment information

Figure 4b summarises the presentation of treatment information on websites. As shown in Figure 5, over half (54%) of the claims within websites had clear, accessible and consistent information. Figure 4b shows that nearly 80% (n = 31, 79%) of websites did not include clear information, and instead used descriptive language and words in place of numbers to quantify magnitude, namely ‘fast’, ‘effective’ and ‘painless’. Five websites (13%) had inconsistent information in the frequently asked questions (FAQs) that was not on the main page, particularly limitations and risks of treatment, need for retention and contradictory claims on treatment times.

### Objectivity of treatment information

Figure 4c summarises the objectivity of the information on websites. All the websites (n = 39) used patient and/or dentist testimonials to promote the appliance. Three websites (8%) used subjective information, such as testimonials and opinions, instead of objective information. Of the websites that included photographs, 14 (56%) of those using professional photographs and all those featuring patient photographs (n = 11) were of poor quality, with limited views that did not adequately represent treatment scope and outcome.

### Substantiation of claims

Figure 4d summarises the substantiation of claims within websites. Excluding websites with no claims, 32% contained exaggerated information about the superiority of materials, technology and treatment time, often in comparison to other appliances. Of the 29 websites that made claims about the material and design, 15 (52%) were exaggerated or improperly cited. Among the 30 websites discussing the treatment process and the 29 websites addressing treatment outcomes, 28 (93%) made reasonable but unsupported claims about the treatment process, while 21 (72%) made similar claims about treatment outcomes, often related to comfort, aesthetics and confidence. Seven websites (25%) made unsubstantiated statements about treatment outcomes, including claims of ‘faster’ or ‘guaranteed’ results. Of the 32 websites that made claims about superiority to comparators, 15 (47%) made these claims without objective evidence. These claims mostly related to faster treatment times and effectiveness compared to conventional braces. As shown in Figure 5, only 4% of all claims across the websites were supported by evidence.

## Comparison of treatment information between appliance categories

**Figure 6.**
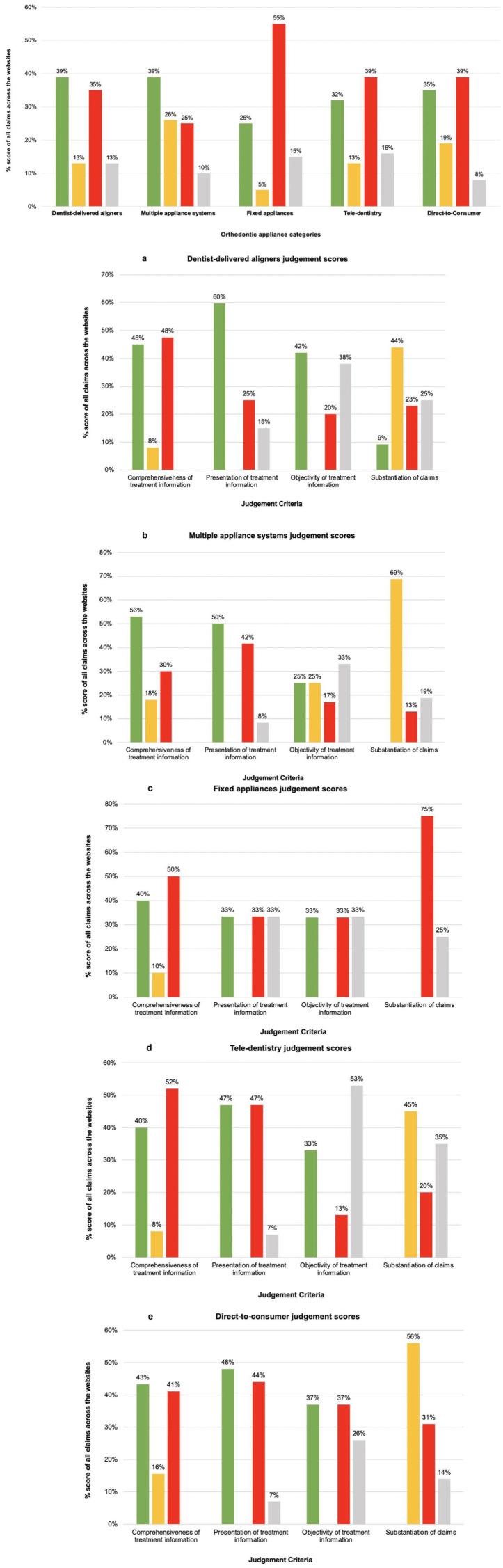
Summary of scores of the five orthodontic appliance categories. The main graph shows the overall comparison of the appliance categories and subgraphs (a–e) present the scores for each appliance category across the four main judgement criteria: (a) dentist-delivered aligners judgement scores; (b) multiple appliance systems judgement scores; (c) fixed appliances judgement scores; (d) tele-dentistry judgement scores; and (e) direct-to-consumer judgement scores. Note: All percentages may not total 100% due to rounding. 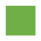
 Complete information/yes/claims are supported by appropriate evidence. 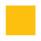
 Partial information/partial/claims are reasonable but not supported by evidence. 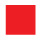
 No information/no/claims are exaggerated or use inappropriate citation. 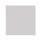
 No FAQ/not used/no claims are made.

Figure 6 summarises the scores for the five orthodontic appliance categories, including the scores for each appliance category across the four main judgement criteria. Dentist-delivered aligners and multiple appliance systems had more substantiated claims with complete information, while fixed appliances, tele-dentistry and DTC websites had more unsubstantiated claims.

### Long-term retention, alternative treatment options and common risks of treatment

At least some information on long-term retention was included on websites of all multiple appliance systems (n = 4), 65% of the dentist-delivered aligners (n = 13), around half of tele-dentistry websites (n = 2) and 78% of the DTC websites (n = 7). No information on alternative treatment options was included on websites of dentist-delivered aligners, multiple appliance systems and fixed appliances, with two tele-dentistry websites (40%) and one DTC website (11%) providing some information about needing fixed treatment for certain cases. Similarly, no information on common risks of treatment was found on websites of dentist-delivered aligners, tele-dentistry and DTC providers. One multiple appliance systems website (25%) and the fixed appliance website included partial information on common risks of treatment (Figure 1).

### Contraindications of treatment and common side effects

No information on treatment contraindications was included on 75% (n = 15) of dentist-delivered websites and 70% (n = 14) did not include any information on common side effects. One dentist-delivered website (5%), two tele-dentistry websites (40%) and one multiple appliance systems website (25%) included complete information on contraindications, explaining that their device could not treat complex orthodontic issues and may be contraindicated if underlying oral health conditions exist, as well as stating age limitations. One multiple appliance systems website (25%) included partial information about the risks, simply stating, ‘*the treatment comes with a calculated risk*’, without supplying further details about what these risks entailed.

### Clarity of information

A total of 70% of dentist-delivered aligner websites (n = 14) lacked clarity of treatment information and used claims about the treatment that could be misleading. For example, ‘*see results [. . .] faster than traditional braces*’, ‘*clear aligners are just as effective and offer the same end results as conventional braces*’, and ‘[can deal with] *complex cases which would not be suitable for therapies with conventional aligners*’. One website claimed it ‘*guarantee*[d] *ideal treatment results*’. Similarly, all multiple appliance systems (n = 4) and tele-dentistry (n = 5) websites included descriptive language, with 78% (n = 7) of DTC websites including terms, such as ‘quick’, ‘fast’ and ‘painless’.

### Substantiation of claims

The fixed appliance website listed over 1000 publications; however, most did not substantiate the advertised benefits, creating a misleading impression of scientific validation. Many references were authored by company affiliates, raising bias concerns, and most were presentations or lectures lacking robust empirical evidence.

Dentist-delivered aligners were the only appliance to include some (9%) substantiated claims about the capability or performance of the appliance. Half of multiple appliance systems websites (n = 2) included unsubstantiated claims; one website claimed it used the ‘*world’s most advanced aligner material*’ and another claimed it was ‘*far more efficient than many other aligners*’. About two-thirds of DTC websites (n = 6, 67%) used unsubstantiated comparisons to other appliances regarding costs and treatment times, without acknowledging that this may depend on treatment objectives. Claims included, ‘*just as effective and offer the same end results as conventional brace*’, ‘*70% cheaper than ordinary braces*’ and ‘*straighten your teeth up to 60% faster than braces*’. Several DTC companies (n = 4, 44%) offered marketing incentives such as ‘money-back guarantees’, payment plans, incentives for referring a friend, promotion on social media or offers to become a brand ambassador or influencer.

## Discussion

### Summary

This study provides the first comprehensive analysis of orthodontic appliance websites against UK advertising standards. Several concerns were identified: most websites used descriptive or exaggerated language and made unsubstantiated claims about treatment objectives, the treatment process and outcomes.

None of the orthodontic appliance websites provided comprehensive information on alternative treatment options or common risks of treatment. Aside from dentist-delivered aligners, no other appliance websites included substantiated claims about their capability or performance. The fixed appliance, tele-dentistry and DTC appliances websites included more exaggerated and unsubstantiated claims, compared to dentist-delivered aligners and multiple appliance systems.

Almost perfect inter-rater reliability was found, indicating strong consistency among raters in their scores (Landis and Koch, 1977).

### Generalisability

The findings are consistent with previous research in this area (Carter and Stokes, 2021; Meade and Dreyer, 2021; Meade et al., 2024), as well as other research, which found the content of orthodontic websites lacked compliance with advertising guidance (Alsaqabi et al., 2023; Meade and Dreyer, 2022; Meade et al., 2023; Parekh and Gill, 2014; Patel and Cobourne, 2015), and advertising by orthodontic manufacturers across social media platforms and in orthodontic journals was of poor quality and lacked high-quality evidence (Alkadhimi et al., 2022; Hameed et al., 2021; Meade et al. 2022). In general, information on the Internet about orthodontic treatment is of poor quality and readability, with an evident need for trustworthy websites to better guide patients (Alpaydın et al., 2021; Arun et al., 2017).

### Interpretation, implications for orthodontic appliance websites and further research

Nearly 80% of websites used descriptive language and made unsubstantiated claims about the speed and comfort of treatment. The ASA identified several commercial companies claiming treatment to be ‘faster’ or ‘less painful’, often in comparison to traditional orthodontic appliances, without providing sufficient substantiating evidence. It stated, ‘*such claims would need to be substantiated with robust documentary evidence in the form of high-quality human clinical trials, which we are yet to see in relation to pain and speed of treatment claims*’ (Advertising Standards Authority, 2018). Risks and limitations of treatment were often omitted in the main body of the website but included in the FAQs. Contradictory information was also found on treatment times in the FAQs compared to that on the main website page. While it is acknowledged that additional information about the treatment process, risks, limitations, and alternative treatments may be provided later in discussions around treatment, this should be provided early enough and in a balanced way to support decision-making. The use of persuasive marketing claims alongside the inadequate upfront discussion of risks in orthodontic-related websites has previously been highlighted as potentially misleading and coercive (Alpaydın et al., 2021; Meade and Dreyer, 2019; Wexler et al., 2020).

Case photographs and patients’ photographs were commonly used on websites, but these were limited in how accurately they portrayed the scope of treatment and likely outcome. The majority had poor-quality images and limited views, for example only showing a frontal intra-oral view or not showing the teeth in occlusion. Images in dentistry as an advertising and marketing tool may be misleading if only the best cases are selected, creating unrealistic patient expectations and dissatisfaction with treatment outcomes (Meira et al., 2021; Simplicio, 2019). Patient and dentist testimonials, which are subjective and at risk of bias, were, in some cases, used in place of objective evidence to support claims made about the treatment process or outcome. This is also consistent with similar research (Holden, 2019; Meade et al., 2024). Coercive marketing strategies were also evident, particularly for the DTC websites, with incentives for referring a friend and social media promotions.

Websites for DTC orthodontic appliances often stated that a dentist devised the treatment plans; however, none mandated a dental assessment before starting orthodontic treatment. Furthermore, the arrangements for monitoring treatment and addressing concerns about treatment progress or outcome were often unclear. Dentists offering orthodontic treatment are responsible for undertaking appropriate examinations, offering all options, working within their scope of practice and being accountable for patient information and treatment (British Orthodontic Society, 2022; General Dental Council, 2021). There is increasing public awareness of potentially misleading information and lack of accountability from some companies, with some being accused of ‘*misleading information* [and] *questionable treatment plans*’ (Canadian Broadcasting Corporation, 2020; NBC News, 2020) and ‘*false advertising*’ (Law360, 2023).

Although there are other guidelines that address health information quality, such as the Health on the Net (HON) Code of Conduct (Boyer et al., 1998), the CAP Code specifically governs advertising standards in the UK to try and ensure that health-related advertisements do not mislead or exaggerate claims. The ASA can investigate and act against advertisements that contravene their code of practice. Focusing on this code is most appropriate, as it directly applies to the claims made within orthodontic company websites.

Responsibility for identifying and managing concerns about advertising standards remains challenging. In 2021, the Care Quality Commission (CQC) decided that those providing aligner treatment should register as providers of ‘regulated activity’. Several companies are now registered and, therefore, are part of the CQC regulatory assessment program. However, the CQC is not able to comment on whether care is effective. Regulators such as the CQC and General Dental Council (GDC) face challenges, because without evidence of harm to the public, they are unable to act. Where harm is reported, the GDC can act against dental registrants and the CQC can address provider issues. Improved transparency and adherence to advertising standards are imperative for informed decision-making in orthodontic care. Ongoing regulatory efforts are essential to address evolving challenges in online orthodontic marketing and uphold public trust.

Within the period of this research, several websites for orthodontic appliances were removed, suggesting the companies are no longer functioning. In summary, 15 DTC and three tele-dentistry companies dissolved; seven new DTC, four new tele-dentistry and nine new dentist-delivered websites emerged; three websites changed their names and two companies merged. This demonstrates the rapid turnover and proliferation of orthodontic companies. Issues with companies dissolving and leaving incomplete treatment have been reported (Daily Mail, 2020). Online forums for discussing at-home aligner appliances have also surfaced, with many customers sharing their stories about being unable to contact companies that are no longer functioning and being left mid-treatment (Smile Prep, 2022). Aside from the loss of time and money, this type of care also puts individuals’ dental health at risk and compromises their orthodontic treatment long-term if they are unable to find a provider able to manage partially complete treatment.

### Limitations

This study provides a comprehensive and systematic assessment of orthodontic appliance promotion on the Internet. However, some limitations should be acknowledged. The ever-changing nature of websites poses a challenge in maintaining accurate data, and we acknowledge the potential for changes between initial data extraction and manuscript finalisation. Despite efforts to create standardised assessment criteria and a robust independent evaluation process, there remains a degree of subjectivity in the judgements made. In addition, the variability in sample sizes across orthodontic appliance categories is acknowledged. Although this may limit the generalisability of findings for individual categories, the study provides valuable insights into the reporting of treatment information across orthodontic appliance websites. Finally, other quality indicators such as readability, website design, navigation and structure were not assessed, but these metrics have been reported elsewhere in detail.

## Conclusion

This study systematically evaluated websites promoting proprietary orthodontic appliances, assessing their adherence to advertising standards and the quality of information provided. Findings revealed significant concerns, with a substantial number of websites failing to convey treatment limitations, risks and alternative options adequately. Descriptive language, subjective content and unsubstantiated claims were prevalent, potentially misleading consumers. Addressing these issues is critical for fostering informed decision-making in orthodontic care.

## Supplemental Material

sj-docx-1-joo-10.1177_14653125251408302 – Supplemental material for Advertising of orthodontic appliances on websites in the UK: Do they comply with advertising standards? A cross-sectional study Supplemental material, sj-docx-1-joo-10.1177_14653125251408302 for Advertising of orthodontic appliances on websites in the UK: Do they comply with advertising standards? A cross-sectional study by Arunika Nehra, Adam Jones and Trevor Hodge in Journal of Orthodontics
