# Supplementary material for: Advertising of orthodontic appliances on websites in the UK: Do they comply with advertising standards? A cross-sectional study
Source: J Orthod. 2026 Feb 26;53(1):45–61. doi: 10.1177/14653125251408302 (PMC12999987; doi:10.1177/14653125251408302)
Supplement: sj-docx-1-joo-10.1177_14653125251408302 – Supplemental material for Advertising of orthodontic appliances on websites in the UK: Do they comply with advertising standards? A cross-sectional study [file sj-docx-1-joo-10.1177_14653125251408302.docx]

**Supplementary Material**

­­­­Supplementary Table**:** Individual rater scores for each website and criterion.

Supplementary Table: Individual rater scores for each website and criterion.

| Website | Main Criteria | Sub Criteria | Rater AN | Rater AJ | Rater SKB | Rater TMH |
| --- | --- | --- | --- | --- | --- | --- |
| 1 | Comprehensiveness of treatment information | Aim of treatment | 1 | 1 | 1 | 1 |
| 1 | Comprehensiveness of treatment information | Mode of action | 1 | 1 | 1 | 1 |
| 1 | Comprehensiveness of treatment information | Scope of treatment | 1 | 1 | 1 | 1 |
| 1 | Comprehensiveness of treatment information | Contraindications | 3 | 3 | 3 | 3 |
| 1 | Comprehensiveness of treatment information | Alternative treatments | 2 | 3 | 3 | 3 |
| 1 | Comprehensiveness of treatment information | Requirements for success | 1 | 1 | 1 | 1 |
| 1 | Comprehensiveness of treatment information | Likely treatment time | 1 | 1 | 1 | 1 |
| 1 | Comprehensiveness of treatment information | Need for long-term retention | 2 | 2 | 2 | 2 |
| 1 | Comprehensiveness of treatment information | Common risks of treatment | 3 | 3 | 3 | 3 |
| 1 | Comprehensiveness of treatment information | Common side effects of treatment | 2 | 2 | 2 | 2 |
| 1 | Presentation of treatment information | Accessibility of information | 1 | 1 | 1 | 1 |
| 1 | Presentation of treatment information | Consistency of information | 1 | 1 | 1 | 1 |
| 1 | Presentation of treatment information | Clarity of information | 1 | 1 | 1 | 1 |
| 1 | Objectivity of treatment information | Use of objective information | 1 | 1 | 1 | 1 |
| 1 | Objectivity of treatment information | Use of professional photographs | 4 | 4 | 4 | 4 |
| 1 | Objectivity of treatment information | Use of patient photographs | 3 | 3 | 3 | 3 |
| 1 | Substantiation of claims | Materials or design | 3 | 3 | 3 | 3 |
| 1 | Substantiation of claims | Treatment process | 2 | 2 | 2 | 2 |
| 1 | Substantiation of claims | Outcome | 1 | 2 | 2 | 2 |
| 1 | Substantiation of claims | Superiority to comparators | 2 | 2 | 2 | 2 |
| 2 | Comprehensiveness of treatment information | Aim of treatment | 1 | 1 | 1 | 1 |
| 2 | Comprehensiveness of treatment information | Mode of action | 1 | 1 | 1 | 1 |
| 2 | Comprehensiveness of treatment information | Scope of treatment | 1 | 1 | 1 | 1 |
| 2 | Comprehensiveness of treatment information | Contraindications | 3 | 3 | 3 | 3 |
| 2 | Comprehensiveness of treatment information | Alternative treatments | 2 | 3 | 3 | 3 |
| 2 | Comprehensiveness of treatment information | Requirements for success | 1 | 1 | 1 | 1 |
| 2 | Comprehensiveness of treatment information | Likely treatment time | 1 | 1 | 1 | 1 |
| 2 | Comprehensiveness of treatment information | Need for long-term retention | 2 | 2 | 2 | 2 |
| 2 | Comprehensiveness of treatment information | Common risks of treatment | 3 | 3 | 3 | 3 |
| 2 | Comprehensiveness of treatment information | Common side effects of treatment | 2 | 2 | 2 | 2 |
| 2 | Presentation of treatment information | Accessibility of information | 1 | 1 | 1 | 1 |
| 2 | Presentation of treatment information | Consistency of information | 1 | 1 | 1 | 1 |
| 2 | Presentation of treatment information | Clarity of information | 1 | 1 | 1 | 1 |
| 2 | Objectivity of treatment information | Use of objective information | 1 | 1 | 1 | 1 |
| 2 | Objectivity of treatment information | Use of professional photographs | 4 | 4 | 4 | 4 |
| 2 | Objectivity of treatment information | Use of patient photographs | 3 | 3 | 3 | 3 |
| 2 | Substantiation of claims | Materials or design | 3 | 3 | 3 | 3 |
| 2 | Substantiation of claims | Treatment process | 2 | 2 | 2 | 2 |
| 2 | Substantiation of claims | Outcome | 1 | 2 | 2 | 2 |
| 2 | Substantiation of claims | Superiority to comparators | 2 | 2 | 2 | 2 |
| 3 | Comprehensiveness of treatment information | Aim of treatment | 1 | 1 | 1 | 1 |
| 3 | Comprehensiveness of treatment information | Mode of action | 1 | 1 | 1 | 1 |
| 3 | Comprehensiveness of treatment information | Scope of treatment | 1 | 2 | 1 | 1 |
| 3 | Comprehensiveness of treatment information | Contraindications | 3 | 3 | 3 | 3 |
| 3 | Comprehensiveness of treatment information | Alternative treatments | 3 | 3 | 3 | 3 |
| 3 | Comprehensiveness of treatment information | Requirements for success | 1 | 2 | 2 | 2 |
| 3 | Comprehensiveness of treatment information | Likely treatment time | 1 | 1 | 1 | 1 |
| 3 | Comprehensiveness of treatment information | Need for long-term retention | 2 | 2 | 2 | 2 |
| 3 | Comprehensiveness of treatment information | Common risks of treatment | 3 | 3 | 3 | 3 |
| 3 | Comprehensiveness of treatment information | Common side effects of treatment | 2 | 2 | 2 | 2 |
| 3 | Presentation of treatment information | Accessibility of information | 1 | 1 | 1 | 1 |
| 3 | Presentation of treatment information | Consistency of information | 1 | 1 | 1 | 1 |
| 3 | Presentation of treatment information | Clarity of information | 3 | 3 | 3 | 3 |
| 3 | Objectivity of treatment information | Use of objective information | 1 | 1 | 1 | 1 |
| 3 | Objectivity of treatment information | Use of professional photographs | 4 | 4 | 4 | 4 |
| 3 | Objectivity of treatment information | Use of patient photographs | 4 | 4 | 4 | 4 |
| 3 | Substantiation of claims | Materials or design | 3 | 3 | 3 | 3 |
| 3 | Substantiation of claims | Treatment process | 2 | 2 | 2 | 2 |
| 3 | Substantiation of claims | Outcome | 3 | 3 | 3 | 3 |
| 3 | Substantiation of claims | Superiority to comparators | 2 | 2 | 2 | 2 |
| 4 | Comprehensiveness of treatment information | Aim of treatment | 2 | 1 | 1 | 1 |
| 4 | Comprehensiveness of treatment information | Mode of action | 1 | 1 | 1 | 1 |
| 4 | Comprehensiveness of treatment information | Scope of treatment | 1 | 1 | 1 | 1 |
| 4 | Comprehensiveness of treatment information | Contraindications | 3 | 3 | 3 | 3 |
| 4 | Comprehensiveness of treatment information | Alternative treatments | 3 | 3 | 3 | 3 |
| 4 | Comprehensiveness of treatment information | Requirements for success | 3 | 3 | 3 | 3 |
| 4 | Comprehensiveness of treatment information | Likely treatment time | 3 | 3 | 3 | 3 |
| 4 | Comprehensiveness of treatment information | Need for long-term retention | 3 | 3 | 3 | 3 |
| 4 | Comprehensiveness of treatment information | Common risks of treatment | 3 | 3 | 3 | 3 |
| 4 | Comprehensiveness of treatment information | Common side effects of treatment | 3 | 3 | 3 | 3 |
| 4 | Presentation of treatment information | Accessibility of information | 1 | 1 | 1 | 1 |
| 4 | Presentation of treatment information | Consistency of information | 4 | 4 | 4 | 4 |
| 4 | Presentation of treatment information | Clarity of information | 3 | 3 | 3 | 3 |
| 4 | Objectivity of treatment information | Use of objective information | 1 | 1 | 1 | 1 |
| 4 | Objectivity of treatment information | Use of professional photographs | 1 | 1 | 1 | 1 |
| 4 | Objectivity of treatment information | Use of patient photographs | 4 | 4 | 4 | 4 |
| 4 | Substantiation of claims | Materials or design | 4 | 4 | 4 | 4 |
| 4 | Substantiation of claims | Treatment process | 4 | 4 | 4 | 4 |
| 4 | Substantiation of claims | Outcome | 2 | 2 | 2 | 2 |
| 4 | Substantiation of claims | Superiority to comparators | 2 | 2 | 2 | 2 |
| 5 | Comprehensiveness of treatment information | Aim of treatment | 1 | 1 | 1 | 1 |
| 5 | Comprehensiveness of treatment information | Mode of action | 1 | 1 | 1 | 1 |
| 5 | Comprehensiveness of treatment information | Scope of treatment | 1 | 2 | 1 | 1 |
| 5 | Comprehensiveness of treatment information | Contraindications | 3 | 3 | 3 | 3 |
| 5 | Comprehensiveness of treatment information | Alternative treatments | 3 | 3 | 3 | 3 |
| 5 | Comprehensiveness of treatment information | Requirements for success | 1 | 2 | 2 | 2 |
| 5 | Comprehensiveness of treatment information | Likely treatment time | 1 | 1 | 1 | 1 |
| 5 | Comprehensiveness of treatment information | Need for long-term retention | 1 | 1 | 1 | 1 |
| 5 | Comprehensiveness of treatment information | Common risks of treatment | 3 | 3 | 3 | 3 |
| 5 | Comprehensiveness of treatment information | Common side effects of treatment | 3 | 3 | 3 | 3 |
| 5 | Presentation of treatment information | Accessibility of information | 1 | 1 | 1 | 1 |
| 5 | Presentation of treatment information | Consistency of information | 1 | 1 | 1 | 1 |
| 5 | Presentation of treatment information | Clarity of information | 3 | 3 | 3 | 3 |
| 5 | Objectivity of treatment information | Use of objective information | 1 | 1 | 1 | 1 |
| 5 | Objectivity of treatment information | Use of professional photographs | 1 | 1 | 1 | 1 |
| 5 | Objectivity of treatment information | Use of patient photographs | 4 | 4 | 4 | 4 |
| 5 | Substantiation of claims | Materials or design | 3 | 3 | 3 | 3 |
| 5 | Substantiation of claims | Treatment process | 2 | 2 | 2 | 2 |
| 5 | Substantiation of claims | Outcome | 3 | 3 | 3 | 3 |
| 5 | Substantiation of claims | Superiority to comparators | 2 | 2 | 2 | 2 |
| 6 | Comprehensiveness of treatment information | Aim of treatment | 1 | 1 | 1 | 1 |
| 6 | Comprehensiveness of treatment information | Mode of action | 3 | 2 | 3 | 3 |
| 6 | Comprehensiveness of treatment information | Scope of treatment | 1 | 1 | 1 | 1 |
| 6 | Comprehensiveness of treatment information | Contraindications | 3 | 3 | 3 | 3 |
| 6 | Comprehensiveness of treatment information | Alternative treatments | 3 | 3 | 3 | 3 |
| 6 | Comprehensiveness of treatment information | Requirements for success | 2 | 2 | 2 | 2 |
| 6 | Comprehensiveness of treatment information | Likely treatment time | 3 | 3 | 3 | 3 |
| 6 | Comprehensiveness of treatment information | Need for long-term retention | 3 | 2 | 3 | 3 |
| 6 | Comprehensiveness of treatment information | Common risks of treatment | 3 | 3 | 3 | 3 |
| 6 | Comprehensiveness of treatment information | Common side effects of treatment | 3 | 3 | 3 | 3 |
| 6 | Presentation of treatment information | Accessibility of information | 1 | 1 | 1 | 1 |
| 6 | Presentation of treatment information | Consistency of information | 4 | 4 | 4 | 4 |
| 6 | Presentation of treatment information | Clarity of information | 3 | 3 | 3 | 3 |
| 6 | Objectivity of treatment information | Use of objective information | 1 | 1 | 1 | 1 |
| 6 | Objectivity of treatment information | Use of professional photographs | 4 | 4 | 4 | 4 |
| 6 | Objectivity of treatment information | Use of patient photographs | 4 | 4 | 4 | 4 |
| 6 | Substantiation of claims | Materials or design | 3 | 3 | 3 | 3 |
| 6 | Substantiation of claims | Treatment process | 4 | 4 | 4 | 4 |
| 6 | Substantiation of claims | Outcome | 2 | 2 | 2 | 2 |
| 6 | Substantiation of claims | Superiority to comparators | 2 | 2 | 2 | 2 |
| 7 | Comprehensiveness of treatment information | Aim of treatment | 1 | 1 | 1 | 1 |
| 7 | Comprehensiveness of treatment information | Mode of action | 1 | 1 | 1 | 1 |
| 7 | Comprehensiveness of treatment information | Scope of treatment | 1 | 1 | 1 | 1 |
| 7 | Comprehensiveness of treatment information | Contraindications | 3 | 3 | 3 | 3 |
| 7 | Comprehensiveness of treatment information | Alternative treatments | 3 | 3 | 3 | 3 |
| 7 | Comprehensiveness of treatment information | Requirements for success | 1 | 1 | 1 | 1 |
| 7 | Comprehensiveness of treatment information | Likely treatment time | 3 | 3 | 3 | 3 |
| 7 | Comprehensiveness of treatment information | Need for long-term retention | 3 | 3 | 3 | 3 |
| 7 | Comprehensiveness of treatment information | Common risks of treatment | 3 | 3 | 3 | 3 |
| 7 | Comprehensiveness of treatment information | Common side effects of treatment | 2 | 2 | 2 | 2 |
| 7 | Presentation of treatment information | Accessibility of information | 1 | 1 | 1 | 1 |
| 7 | Presentation of treatment information | Consistency of information | 4 | 4 | 4 | 4 |
| 7 | Presentation of treatment information | Clarity of information | 1 | 1 | 1 | 1 |
| 7 | Objectivity of treatment information | Use of objective information | 1 | 1 | 1 | 1 |
| 7 | Objectivity of treatment information | Use of professional photographs | 3 | 3 | 3 | 3 |
| 7 | Objectivity of treatment information | Use of patient photographs | 4 | 4 | 4 | 4 |
| 7 | Substantiation of claims | Materials or design | 2 | 2 | 2 | 2 |
| 7 | Substantiation of claims | Treatment process | 2 | 2 | 2 | 2 |
| 7 | Substantiation of claims | Outcome | 2 | 2 | 2 | 2 |
| 7 | Substantiation of claims | Superiority to comparators | 2 | 2 | 2 | 2 |
| 8 | Comprehensiveness of treatment information | Aim of treatment | 1 | 1 | 1 | 1 |
| 8 | Comprehensiveness of treatment information | Mode of action | 1 | 1 | 1 | 1 |
| 8 | Comprehensiveness of treatment information | Scope of treatment | 3 | 3 | 3 | 3 |
| 8 | Comprehensiveness of treatment information | Contraindications | 3 | 3 | 3 | 3 |
| 8 | Comprehensiveness of treatment information | Alternative treatments | 3 | 3 | 3 | 3 |
| 8 | Comprehensiveness of treatment information | Requirements for success | 3 | 3 | 3 | 3 |
| 8 | Comprehensiveness of treatment information | Likely treatment time | 1 | 1 | 1 | 1 |
| 8 | Comprehensiveness of treatment information | Need for long-term retention | 3 | 3 | 3 | 3 |
| 8 | Comprehensiveness of treatment information | Common risks of treatment | 3 | 3 | 3 | 3 |
| 8 | Comprehensiveness of treatment information | Common side effects of treatment | 1 | 1 | 1 | 1 |
| 8 | Presentation of treatment information | Accessibility of information | 1 | 1 | 1 | 1 |
| 8 | Presentation of treatment information | Consistency of information | 1 | 1 | 1 | 1 |
| 8 | Presentation of treatment information | Clarity of information | 1 | 1 | 1 | 1 |
| 8 | Objectivity of treatment information | Use of objective information | 1 | 1 | 1 | 1 |
| 8 | Objectivity of treatment information | Use of professional photographs | 3 | 3 | 3 | 3 |
| 8 | Objectivity of treatment information | Use of patient photographs | 4 | 4 | 4 | 4 |
| 8 | Substantiation of claims | Materials or design | 2 | 2 | 2 | 2 |
| 8 | Substantiation of claims | Treatment process | 4 | 4 | 4 | 4 |
| 8 | Substantiation of claims | Outcome | 2 | 2 | 2 | 2 |
| 8 | Substantiation of claims | Superiority to comparators | 4 | 4 | 4 | 4 |
| 9 | Comprehensiveness of treatment information | Aim of treatment | 3 | 3 | 3 | 3 |
| 9 | Comprehensiveness of treatment information | Mode of action | 1 | 1 | 1 | 1 |
| 9 | Comprehensiveness of treatment information | Scope of treatment | 1 | 1 | 1 | 1 |
| 9 | Comprehensiveness of treatment information | Contraindications | 3 | 3 | 3 | 3 |
| 9 | Comprehensiveness of treatment information | Alternative treatments | 3 | 3 | 3 | 3 |
| 9 | Comprehensiveness of treatment information | Requirements for success | 2 | 2 | 2 | 2 |
| 9 | Comprehensiveness of treatment information | Likely treatment time | 1 | 1 | 1 | 1 |
| 9 | Comprehensiveness of treatment information | Need for long-term retention | 1 | 1 | 1 | 1 |
| 9 | Comprehensiveness of treatment information | Common risks of treatment | 3 | 3 | 3 | 3 |
| 9 | Comprehensiveness of treatment information | Common side effects of treatment | 3 | 3 | 3 | 3 |
| 9 | Presentation of treatment information | Accessibility of information | 1 | 1 | 1 | 1 |
| 9 | Presentation of treatment information | Consistency of information | 4 | 4 | 4 | 4 |
| 9 | Presentation of treatment information | Clarity of information | 1 | 1 | 1 | 1 |
| 9 | Objectivity of treatment information | Use of objective information | 1 | 1 | 1 | 1 |
| 9 | Objectivity of treatment information | Use of professional photographs | 3 | 3 | 3 | 3 |
| 9 | Objectivity of treatment information | Use of patient photographs | 4 | 4 | 4 | 4 |
| 9 | Substantiation of claims | Materials or design | 1 | 1 | 1 | 1 |
| 9 | Substantiation of claims | Treatment process | 2 | 2 | 2 | 2 |
| 9 | Substantiation of claims | Outcome | 2 | 2 | 2 | 2 |
| 9 | Substantiation of claims | Superiority to comparators | 3 | 3 | 3 | 3 |
| 10 | Comprehensiveness of treatment information | Aim of treatment | 1 | 1 | 1 | 1 |
| 10 | Comprehensiveness of treatment information | Mode of action | 1 | 1 | 1 | 1 |
| 10 | Comprehensiveness of treatment information | Scope of treatment | 1 | 1 | 1 | 1 |
| 10 | Comprehensiveness of treatment information | Contraindications | 3 | 3 | 3 | 3 |
| 10 | Comprehensiveness of treatment information | Alternative treatments | 3 | 3 | 3 | 3 |
| 10 | Comprehensiveness of treatment information | Requirements for success | 3 | 3 | 3 | 3 |
| 10 | Comprehensiveness of treatment information | Likely treatment time | 1 | 1 | 1 | 1 |
| 10 | Comprehensiveness of treatment information | Need for long-term retention | 2 | 2 | 2 | 2 |
| 10 | Comprehensiveness of treatment information | Common risks of treatment | 3 | 3 | 3 | 3 |
| 10 | Comprehensiveness of treatment information | Common side effects of treatment | 3 | 3 | 3 | 3 |
| 10 | Presentation of treatment information | Accessibility of information | 1 | 1 | 1 | 1 |
| 10 | Presentation of treatment information | Consistency of information | 1 | 1 | 1 | 1 |
| 10 | Presentation of treatment information | Clarity of information | 3 | 3 | 3 | 3 |
| 10 | Objectivity of treatment information | Use of objective information | 1 | 1 | 1 | 1 |
| 10 | Objectivity of treatment information | Use of professional photographs | 3 | 3 | 3 | 3 |
| 10 | Objectivity of treatment information | Use of patient photographs | 4 | 4 | 4 | 4 |
| 10 | Substantiation of claims | Materials or design | 4 | 4 | 4 | 4 |
| 10 | Substantiation of claims | Treatment process | 2 | 2 | 2 | 2 |
| 10 | Substantiation of claims | Outcome | 4 | 4 | 4 | 4 |
| 10 | Substantiation of claims | Superiority to comparators | 3 | 3 | 3 | 3 |
| 11 | Comprehensiveness of treatment information | Aim of treatment | 1 | 1 | 1 | 1 |
| 11 | Comprehensiveness of treatment information | Mode of action | 1 | 1 | 1 | 1 |
| 11 | Comprehensiveness of treatment information | Scope of treatment | 1 | 1 | 1 | 1 |
| 11 | Comprehensiveness of treatment information | Contraindications | 3 | 3 | 3 | 3 |
| 11 | Comprehensiveness of treatment information | Alternative treatments | 3 | 3 | 3 | 3 |
| 11 | Comprehensiveness of treatment information | Requirements for success | 1 | 1 | 1 | 1 |
| 11 | Comprehensiveness of treatment information | Likely treatment time | 1 | 1 | 1 | 1 |
| 11 | Comprehensiveness of treatment information | Need for long-term retention | 1 | 1 | 1 | 1 |
| 11 | Comprehensiveness of treatment information | Common risks of treatment | 3 | 3 | 3 | 3 |
| 11 | Comprehensiveness of treatment information | Common side effects of treatment | 1 | 1 | 1 | 1 |
| 11 | Presentation of treatment information | Accessibility of information | 1 | 1 | 1 | 1 |
| 11 | Presentation of treatment information | Consistency of information | 1 | 1 | 1 | 1 |
| 11 | Presentation of treatment information | Clarity of information | 3 | 3 | 3 | 3 |
| 11 | Objectivity of treatment information | Use of objective information | 1 | 1 | 1 | 1 |
| 11 | Objectivity of treatment information | Use of professional photographs | 4 | 4 | 4 | 4 |
| 11 | Objectivity of treatment information | Use of patient photographs | 4 | 4 | 4 | 4 |
| 11 | Substantiation of claims | Materials or design | 4 | 4 | 4 | 4 |
| 11 | Substantiation of claims | Treatment process | 2 | 2 | 2 | 2 |
| 11 | Substantiation of claims | Outcome | 4 | 4 | 4 | 4 |
| 11 | Substantiation of claims | Superiority to comparators | 4 | 4 | 4 | 4 |
| 12 | Comprehensiveness of treatment information | Aim of treatment | 3 | 3 | 3 | 3 |
| 12 | Comprehensiveness of treatment information | Mode of action | 1 | 1 | 1 | 1 |
| 12 | Comprehensiveness of treatment information | Scope of treatment | 1 | 1 | 1 | 1 |
| 12 | Comprehensiveness of treatment information | Contraindications | 3 | 3 | 3 | 3 |
| 12 | Comprehensiveness of treatment information | Alternative treatments | 3 | 3 | 3 | 3 |
| 12 | Comprehensiveness of treatment information | Requirements for success | 3 | 3 | 3 | 3 |
| 12 | Comprehensiveness of treatment information | Likely treatment time | 1 | 1 | 1 | 1 |
| 12 | Comprehensiveness of treatment information | Need for long-term retention | 1 | 1 | 1 | 1 |
| 12 | Comprehensiveness of treatment information | Common risks of treatment | 3 | 3 | 3 | 3 |
| 12 | Comprehensiveness of treatment information | Common side effects of treatment | 3 | 3 | 3 | 3 |
| 12 | Presentation of treatment information | Accessibility of information | 1 | 1 | 1 | 1 |
| 12 | Presentation of treatment information | Consistency of information | 1 | 1 | 1 | 1 |
| 12 | Presentation of treatment information | Clarity of information | 3 | 3 | 3 | 3 |
| 12 | Objectivity of treatment information | Use of objective information | 3 | 3 | 3 | 3 |
| 12 | Objectivity of treatment information | Use of professional photographs | 4 | 4 | 4 | 4 |
| 12 | Objectivity of treatment information | Use of patient photographs | 3 | 3 | 3 | 3 |
| 12 | Substantiation of claims | Materials or design | 3 | 3 | 3 | 3 |
| 12 | Substantiation of claims | Treatment process | 2 | 2 | 2 | 2 |
| 12 | Substantiation of claims | Outcome | 2 | 2 | 2 | 2 |
| 12 | Substantiation of claims | Superiority to comparators | 3 | 3 | 3 | 3 |
| 13 | Comprehensiveness of treatment information | Aim of treatment | 1 | 1 | 1 | 1 |
| 13 | Comprehensiveness of treatment information | Mode of action | 1 | 1 | 1 | 1 |
| 13 | Comprehensiveness of treatment information | Scope of treatment | 1 | 1 | 1 | 1 |
| 13 | Comprehensiveness of treatment information | Contraindications | 2 | 3 | 2 | 2 |
| 13 | Comprehensiveness of treatment information | Alternative treatments | 3 | 3 | 3 | 3 |
| 13 | Comprehensiveness of treatment information | Requirements for success | 1 | 1 | 1 | 1 |
| 13 | Comprehensiveness of treatment information | Likely treatment time | 1 | 1 | 1 | 1 |
| 13 | Comprehensiveness of treatment information | Need for long-term retention | 1 | 1 | 1 | 1 |
| 13 | Comprehensiveness of treatment information | Common risks of treatment | 3 | 3 | 3 | 3 |
| 13 | Comprehensiveness of treatment information | Common side effects of treatment | 3 | 3 | 3 | 3 |
| 13 | Presentation of treatment information | Accessibility of information | 1 | 1 | 1 | 1 |
| 13 | Presentation of treatment information | Consistency of information | 4 | 4 | 4 | 4 |
| 13 | Presentation of treatment information | Clarity of information | 3 | 3 | 3 | 3 |
| 13 | Objectivity of treatment information | Use of objective information | 1 | 1 | 1 | 1 |
| 13 | Objectivity of treatment information | Use of professional photographs | 1 | 1 | 1 | 1 |
| 13 | Objectivity of treatment information | Use of patient photographs | 4 | 4 | 4 | 4 |
| 13 | Substantiation of claims | Materials or design | 3 | 3 | 3 | 3 |
| 13 | Substantiation of claims | Treatment process | 4 | 4 | 4 | 4 |
| 13 | Substantiation of claims | Outcome | 2 | 2 | 2 | 2 |
| 13 | Substantiation of claims | Superiority to comparators | 2 | 2 | 2 | 2 |
| 14 | Comprehensiveness of treatment information | Aim of treatment | 1 | 1 | 1 | 1 |
| 14 | Comprehensiveness of treatment information | Mode of action | 1 | 1 | 1 | 1 |
| 14 | Comprehensiveness of treatment information | Scope of treatment | 1 | 1 | 1 | 1 |
| 14 | Comprehensiveness of treatment information | Contraindications | 2 | 3 | 2 | 2 |
| 14 | Comprehensiveness of treatment information | Alternative treatments | 3 | 3 | 3 | 3 |
| 14 | Comprehensiveness of treatment information | Requirements for success | 1 | 2 | 1 | 1 |
| 14 | Comprehensiveness of treatment information | Likely treatment time | 1 | 1 | 1 | 1 |
| 14 | Comprehensiveness of treatment information | Need for long-term retention | 1 | 1 | 1 | 1 |
| 14 | Comprehensiveness of treatment information | Common risks of treatment | 3 | 3 | 3 | 3 |
| 14 | Comprehensiveness of treatment information | Common side effects of treatment | 3 | 3 | 3 | 3 |
| 14 | Presentation of treatment information | Accessibility of information | 1 | 1 | 1 | 1 |
| 14 | Presentation of treatment information | Consistency of information | 4 | 4 | 4 | 4 |
| 14 | Presentation of treatment information | Clarity of information | 3 | 3 | 3 | 3 |
| 14 | Objectivity of treatment information | Use of objective information | 1 | 1 | 1 | 1 |
| 14 | Objectivity of treatment information | Use of professional photographs | 1 | 1 | 1 | 1 |
| 14 | Objectivity of treatment information | Use of patient photographs | 4 | 4 | 4 | 4 |
| 14 | Substantiation of claims | Materials or design | 4 | 4 | 4 | 4 |
| 14 | Substantiation of claims | Treatment process | 2 | 2 | 2 | 2 |
| 14 | Substantiation of claims | Outcome | 2 | 2 | 2 | 2 |
| 14 | Substantiation of claims | Superiority to comparators | 4 | 4 | 4 | 4 |
| 15 | Comprehensiveness of treatment information | Aim of treatment | 1 | 1 | 1 | 1 |
| 15 | Comprehensiveness of treatment information | Mode of action | 1 | 1 | 1 | 1 |
| 15 | Comprehensiveness of treatment information | Scope of treatment | 1 | 1 | 1 | 1 |
| 15 | Comprehensiveness of treatment information | Contraindications | 2 | 3 | 2 | 2 |
| 15 | Comprehensiveness of treatment information | Alternative treatments | 3 | 3 | 3 | 3 |
| 15 | Comprehensiveness of treatment information | Requirements for success | 1 | 1 | 1 | 1 |
| 15 | Comprehensiveness of treatment information | Likely treatment time | 1 | 1 | 1 | 1 |
| 15 | Comprehensiveness of treatment information | Need for long-term retention | 1 | 1 | 1 | 1 |
| 15 | Comprehensiveness of treatment information | Common risks of treatment | 3 | 3 | 3 | 3 |
| 15 | Comprehensiveness of treatment information | Common side effects of treatment | 3 | 3 | 3 | 3 |
| 15 | Presentation of treatment information | Accessibility of information | 1 | 1 | 1 | 1 |
| 15 | Presentation of treatment information | Consistency of information | 1 | 1 | 1 | 1 |
| 15 | Presentation of treatment information | Clarity of information | 3 | 3 | 3 | 3 |
| 15 | Objectivity of treatment information | Use of objective information | 2 | 1 | 1 | 1 |
| 15 | Objectivity of treatment information | Use of professional photographs | 4 | 4 | 4 | 4 |
| 15 | Objectivity of treatment information | Use of patient photographs | 3 | 3 | 3 | 3 |
| 15 | Substantiation of claims | Materials or design | 1 | 1 | 1 | 1 |
| 15 | Substantiation of claims | Treatment process | 2 | 2 | 2 | 2 |
| 15 | Substantiation of claims | Outcome | 1 | 1 | 1 | 1 |
| 15 | Substantiation of claims | Superiority to comparators | 1 | 1 | 1 | 1 |
| 16 | Comprehensiveness of treatment information | Aim of treatment | 1 | 1 | 1 | 1 |
| 16 | Comprehensiveness of treatment information | Mode of action | 1 | 1 | 1 | 1 |
| 16 | Comprehensiveness of treatment information | Scope of treatment | 1 | 1 | 1 | 1 |
| 16 | Comprehensiveness of treatment information | Contraindications | 3 | 3 | 3 | 3 |
| 16 | Comprehensiveness of treatment information | Alternative treatments | 3 | 3 | 3 | 3 |
| 16 | Comprehensiveness of treatment information | Requirements for success | 3 | 3 | 3 | 3 |
| 16 | Comprehensiveness of treatment information | Likely treatment time | 3 | 3 | 3 | 3 |
| 16 | Comprehensiveness of treatment information | Need for long-term retention | 3 | 3 | 3 | 3 |
| 16 | Comprehensiveness of treatment information | Common risks of treatment | 3 | 3 | 3 | 3 |
| 16 | Comprehensiveness of treatment information | Common side effects of treatment | 3 | 3 | 3 | 3 |
| 16 | Presentation of treatment information | Accessibility of information | 3 | 3 | 3 | 3 |
| 16 | Presentation of treatment information | Consistency of information | 4 | 4 | 4 | 4 |
| 16 | Presentation of treatment information | Clarity of information | 3 | 3 | 3 | 3 |
| 16 | Objectivity of treatment information | Use of objective information | 1 | 1 | 1 | 1 |
| 16 | Objectivity of treatment information | Use of professional photographs | 3 | 3 | 3 | 3 |
| 16 | Objectivity of treatment information | Use of patient photographs | 4 | 4 | 4 | 4 |
| 16 | Substantiation of claims | Materials or design | 2 | 2 | 2 | 2 |
| 16 | Substantiation of claims | Treatment process | 3 | 3 | 3 | 3 |
| 16 | Substantiation of claims | Outcome | 3 | 3 | 3 | 3 |
| 16 | Substantiation of claims | Superiority to comparators | 3 | 3 | 3 | 3 |
| 17 | Comprehensiveness of treatment information | Aim of treatment | 1 | 1 | 1 | 1 |
| 17 | Comprehensiveness of treatment information | Mode of action | 1 | 1 | 1 | 1 |
| 17 | Comprehensiveness of treatment information | Scope of treatment | 2 | 1 | 2 | 2 |
| 17 | Comprehensiveness of treatment information | Contraindications | 1 | 2 | 2 | 2 |
| 17 | Comprehensiveness of treatment information | Alternative treatments | 3 | 3 | 3 | 3 |
| 17 | Comprehensiveness of treatment information | Requirements for success | 1 | 1 | 1 | 1 |
| 17 | Comprehensiveness of treatment information | Likely treatment time | 1 | 1 | 1 | 1 |
| 17 | Comprehensiveness of treatment information | Need for long-term retention | 1 | 1 | 1 | 1 |
| 17 | Comprehensiveness of treatment information | Common risks of treatment | 3 | 3 | 3 | 3 |
| 17 | Comprehensiveness of treatment information | Common side effects of treatment | 3 | 3 | 3 | 3 |
| 17 | Presentation of treatment information | Accessibility of information | 1 | 1 | 1 | 1 |
| 17 | Presentation of treatment information | Consistency of information | 1 | 1 | 1 | 1 |
| 17 | Presentation of treatment information | Clarity of information | 3 | 3 | 3 | 3 |
| 17 | Objectivity of treatment information | Use of objective information | 1 | 1 | 1 | 1 |
| 17 | Objectivity of treatment information | Use of professional photographs | 3 | 3 | 3 | 3 |
| 17 | Objectivity of treatment information | Use of patient photographs | 4 | 4 | 4 | 4 |
| 17 | Substantiation of claims | Materials or design | 4 | 4 | 4 | 4 |
| 17 | Substantiation of claims | Treatment process | 2 | 2 | 2 | 2 |
| 17 | Substantiation of claims | Outcome | 4 | 4 | 4 | 4 |
| 17 | Substantiation of claims | Superiority to comparators | 2 | 2 | 2 | 2 |
| 18 | Comprehensiveness of treatment information | Aim of treatment | 1 | 1 | 1 | 1 |
| 18 | Comprehensiveness of treatment information | Mode of action | 1 | 1 | 1 | 1 |
| 18 | Comprehensiveness of treatment information | Scope of treatment | 1 | 1 | 1 | 1 |
| 18 | Comprehensiveness of treatment information | Contraindications | 1 | 1 | 1 | 1 |
| 18 | Comprehensiveness of treatment information | Alternative treatments | 3 | 3 | 3 | 3 |
| 18 | Comprehensiveness of treatment information | Requirements for success | 2 | 2 | 2 | 2 |
| 18 | Comprehensiveness of treatment information | Likely treatment time | 3 | 2 | 3 | 3 |
| 18 | Comprehensiveness of treatment information | Need for long-term retention | 3 | 3 | 3 | 3 |
| 18 | Comprehensiveness of treatment information | Common risks of treatment | 3 | 3 | 3 | 3 |
| 18 | Comprehensiveness of treatment information | Common side effects of treatment | 1 | 1 | 1 | 1 |
| 18 | Presentation of treatment information | Accessibility of information | 1 | 1 | 1 | 1 |
| 18 | Presentation of treatment information | Consistency of information | 1 | 1 | 1 | 1 |
| 18 | Presentation of treatment information | Clarity of information | 3 | 3 | 3 | 3 |
| 18 | Objectivity of treatment information | Use of objective information | 1 | 1 | 1 | 1 |
| 18 | Objectivity of treatment information | Use of professional photographs | 3 | 3 | 3 | 3 |
| 18 | Objectivity of treatment information | Use of patient photographs | 4 | 4 | 4 | 4 |
| 18 | Substantiation of claims | Materials or design | 1 | 1 | 1 | 1 |
| 18 | Substantiation of claims | Treatment process | 1 | 1 | 1 | 1 |
| 18 | Substantiation of claims | Outcome | 4 | 4 | 4 | 4 |
| 18 | Substantiation of claims | Superiority to comparators | 1 | 1 | 1 | 1 |
| 19 | Comprehensiveness of treatment information | Aim of treatment | 1 | 1 | 1 | 1 |
| 19 | Comprehensiveness of treatment information | Mode of action | 1 | 1 | 1 | 1 |
| 19 | Comprehensiveness of treatment information | Scope of treatment | 1 | 1 | 1 | 1 |
| 19 | Comprehensiveness of treatment information | Contraindications | 3 | 3 | 3 | 3 |
| 19 | Comprehensiveness of treatment information | Alternative treatments | 3 | 3 | 3 | 3 |
| 19 | Comprehensiveness of treatment information | Requirements for success | 1 | 1 | 1 | 1 |
| 19 | Comprehensiveness of treatment information | Likely treatment time | 3 | 3 | 3 | 3 |
| 19 | Comprehensiveness of treatment information | Need for long-term retention | 1 | 1 | 1 | 1 |
| 19 | Comprehensiveness of treatment information | Common risks of treatment | 3 | 3 | 3 | 3 |
| 19 | Comprehensiveness of treatment information | Common side effects of treatment | 3 | 3 | 3 | 3 |
| 19 | Presentation of treatment information | Accessibility of information | 1 | 1 | 1 | 1 |
| 19 | Presentation of treatment information | Consistency of information | 4 | 4 | 4 | 4 |
| 19 | Presentation of treatment information | Clarity of information | 1 | 1 | 1 | 1 |
| 19 | Objectivity of treatment information | Use of objective information | 1 | 1 | 1 | 1 |
| 19 | Objectivity of treatment information | Use of professional photographs | 3 | 3 | 3 | 3 |
| 19 | Objectivity of treatment information | Use of patient photographs | 4 | 4 | 4 | 4 |
| 19 | Substantiation of claims | Materials or design | 1 | 1 | 1 | 1 |
| 19 | Substantiation of claims | Treatment process | 2 | 2 | 2 | 2 |
| 19 | Substantiation of claims | Outcome | 2 | 2 | 2 | 2 |
| 19 | Substantiation of claims | Superiority to comparators | 4 | 4 | 4 | 4 |
| 20 | Comprehensiveness of treatment information | Aim of treatment | 2 | 1 | 1 | 1 |
| 20 | Comprehensiveness of treatment information | Mode of action | 1 | 1 | 1 | 1 |
| 20 | Comprehensiveness of treatment information | Scope of treatment | 1 | 1 | 1 | 1 |
| 20 | Comprehensiveness of treatment information | Contraindications | 3 | 3 | 3 | 3 |
| 20 | Comprehensiveness of treatment information | Alternative treatments | 3 | 3 | 3 | 3 |
| 20 | Comprehensiveness of treatment information | Requirements for success | 1 | 1 | 1 | 1 |
| 20 | Comprehensiveness of treatment information | Likely treatment time | 3 | 3 | 3 | 3 |
| 20 | Comprehensiveness of treatment information | Need for long-term retention | 1 | 1 | 1 | 1 |
| 20 | Comprehensiveness of treatment information | Common risks of treatment | 3 | 3 | 3 | 3 |
| 20 | Comprehensiveness of treatment information | Common side effects of treatment | 3 | 3 | 3 | 3 |
| 20 | Presentation of treatment information | Accessibility of information | 1 | 1 | 1 | 1 |
| 20 | Presentation of treatment information | Consistency of information | 1 | 1 | 1 | 1 |
| 20 | Presentation of treatment information | Clarity of information | 3 | 3 | 3 | 3 |
| 20 | Objectivity of treatment information | Use of objective information | 1 | 1 | 1 | 1 |
| 20 | Objectivity of treatment information | Use of professional photographs | 1 | 1 | 1 | 1 |
| 20 | Objectivity of treatment information | Use of patient photographs | 4 | 4 | 4 | 4 |
| 20 | Substantiation of claims | Materials or design | 3 | 3 | 3 | 3 |
| 20 | Substantiation of claims | Treatment process | 2 | 2 | 2 | 2 |
| 20 | Substantiation of claims | Outcome | 4 | 4 | 4 | 4 |
| 20 | Substantiation of claims | Superiority to comparators | 4 | 4 | 4 | 4 |
| 21 | Comprehensiveness of treatment information | Aim of treatment | 1 | 1 | 1 | 1 |
| 21 | Comprehensiveness of treatment information | Mode of action | 1 | 1 | 1 | 1 |
| 21 | Comprehensiveness of treatment information | Scope of treatment | 1 | 1 | 1 | 1 |
| 21 | Comprehensiveness of treatment information | Contraindications | 1 | 1 | 1 | 1 |
| 21 | Comprehensiveness of treatment information | Alternative treatments | 3 | 3 | 3 | 3 |
| 21 | Comprehensiveness of treatment information | Requirements for success | 1 | 1 | 1 | 1 |
| 21 | Comprehensiveness of treatment information | Likely treatment time | 1 | 2 | 1 | 1 |
| 21 | Comprehensiveness of treatment information | Need for long-term retention | 1 | 1 | 1 | 1 |
| 21 | Comprehensiveness of treatment information | Common risks of treatment | 3 | 3 | 3 | 3 |
| 21 | Comprehensiveness of treatment information | Common side effects of treatment | 2 | 2 | 2 | 2 |
| 21 | Presentation of treatment information | Accessibility of information | 1 | 1 | 1 | 1 |
| 21 | Presentation of treatment information | Consistency of information | 3 | 3 | 3 | 3 |
| 21 | Presentation of treatment information | Clarity of information | 3 | 3 | 3 | 3 |
| 21 | Objectivity of treatment information | Use of objective information | 2 | 1 | 1 | 1 |
| 21 | Objectivity of treatment information | Use of professional photographs | 2 | 2 | 2 | 2 |
| 21 | Objectivity of treatment information | Use of patient photographs | 4 | 4 | 4 | 4 |
| 21 | Substantiation of claims | Materials or design | 4 | 4 | 4 | 4 |
| 21 | Substantiation of claims | Treatment process | 2 | 2 | 2 | 2 |
| 21 | Substantiation of claims | Outcome | 2 | 2 | 2 | 2 |
| 21 | Substantiation of claims | Superiority to comparators | 2 | 2 | 2 | 2 |
| 22 | Comprehensiveness of treatment information | Aim of treatment | 1 | 1 | 1 | 1 |
| 22 | Comprehensiveness of treatment information | Mode of action | 1 | 1 | 1 | 1 |
| 22 | Comprehensiveness of treatment information | Scope of treatment | 2 | 1 | 2 | 2 |
| 22 | Comprehensiveness of treatment information | Contraindications | 3 | 3 | 3 | 3 |
| 22 | Comprehensiveness of treatment information | Alternative treatments | 3 | 3 | 3 | 3 |
| 22 | Comprehensiveness of treatment information | Requirements for success | 1 | 2 | 2 | 2 |
| 22 | Comprehensiveness of treatment information | Likely treatment time | 1 | 1 | 1 | 1 |
| 22 | Comprehensiveness of treatment information | Need for long-term retention | 1 | 1 | 1 | 1 |
| 22 | Comprehensiveness of treatment information | Common risks of treatment | 2 | 1 | 2 | 2 |
| 22 | Comprehensiveness of treatment information | Common side effects of treatment | 3 | 3 | 3 | 3 |
| 22 | Presentation of treatment information | Accessibility of information | 1 | 1 | 1 | 1 |
| 22 | Presentation of treatment information | Consistency of information | 4 | 4 | 4 | 4 |
| 22 | Presentation of treatment information | Clarity of information | 3 | 3 | 3 | 3 |
| 22 | Objectivity of treatment information | Use of objective information | 1 | 1 | 1 | 1 |
| 22 | Objectivity of treatment information | Use of professional photographs | 2 | 2 | 2 | 2 |
| 22 | Objectivity of treatment information | Use of patient photographs | 4 | 4 | 4 | 4 |
| 22 | Substantiation of claims | Materials or design | 4 | 4 | 4 | 4 |
| 22 | Substantiation of claims | Treatment process | 2 | 2 | 2 | 2 |
| 22 | Substantiation of claims | Outcome | 2 | 2 | 2 | 2 |
| 22 | Substantiation of claims | Superiority to comparators | 3 | 3 | 3 | 3 |
| 23 | Comprehensiveness of treatment information | Aim of treatment | 1 | 1 | 1 | 1 |
| 23 | Comprehensiveness of treatment information | Mode of action | 3 | 3 | 3 | 3 |
| 23 | Comprehensiveness of treatment information | Scope of treatment | 1 | 1 | 1 | 1 |
| 23 | Comprehensiveness of treatment information | Contraindications | 3 | 3 | 3 | 3 |
| 23 | Comprehensiveness of treatment information | Alternative treatments | 3 | 3 | 3 | 3 |
| 23 | Comprehensiveness of treatment information | Requirements for success | 1 | 1 | 1 | 1 |
| 23 | Comprehensiveness of treatment information | Likely treatment time | 1 | 1 | 1 | 1 |
| 23 | Comprehensiveness of treatment information | Need for long-term retention | 1 | 1 | 1 | 1 |
| 23 | Comprehensiveness of treatment information | Common risks of treatment | 3 | 3 | 3 | 3 |
| 23 | Comprehensiveness of treatment information | Common side effects of treatment | 2 | 2 | 2 | 2 |
| 23 | Presentation of treatment information | Accessibility of information | 1 | 1 | 1 | 1 |
| 23 | Presentation of treatment information | Consistency of information | 1 | 1 | 1 | 1 |
| 23 | Presentation of treatment information | Clarity of information | 3 | 3 | 3 | 3 |
| 23 | Objectivity of treatment information | Use of objective information | 1 | 1 | 1 | 1 |
| 23 | Objectivity of treatment information | Use of professional photographs | 3 | 3 | 3 | 3 |
| 23 | Objectivity of treatment information | Use of patient photographs | 4 | 4 | 4 | 4 |
| 23 | Substantiation of claims | Materials or design | 2 | 2 | 2 | 2 |
| 23 | Substantiation of claims | Treatment process | 2 | 2 | 2 | 2 |
| 23 | Substantiation of claims | Outcome | 2 | 2 | 2 | 2 |
| 23 | Substantiation of claims | Superiority to comparators | 2 | 2 | 2 | 2 |
| 24 | Comprehensiveness of treatment information | Aim of treatment | 1 | 1 | 1 | 1 |
| 24 | Comprehensiveness of treatment information | Mode of action | 1 | 1 | 1 | 1 |
| 24 | Comprehensiveness of treatment information | Scope of treatment | 1 | 1 | 1 | 1 |
| 24 | Comprehensiveness of treatment information | Contraindications | 3 | 3 | 3 | 3 |
| 24 | Comprehensiveness of treatment information | Alternative treatments | 3 | 3 | 3 | 3 |
| 24 | Comprehensiveness of treatment information | Requirements for success | 2 | 1 | 2 | 2 |
| 24 | Comprehensiveness of treatment information | Likely treatment time | 1 | 1 | 1 | 1 |
| 24 | Comprehensiveness of treatment information | Need for long-term retention | 1 | 1 | 1 | 1 |
| 24 | Comprehensiveness of treatment information | Common risks of treatment | 3 | 3 | 3 | 3 |
| 24 | Comprehensiveness of treatment information | Common side effects of treatment | 2 | 2 | 2 | 2 |
| 24 | Presentation of treatment information | Accessibility of information | 1 | 1 | 1 | 1 |
| 24 | Presentation of treatment information | Consistency of information | 1 | 1 | 1 | 1 |
| 24 | Presentation of treatment information | Clarity of information | 3 | 3 | 3 | 3 |
| 24 | Objectivity of treatment information | Use of objective information | 3 | 3 | 3 | 3 |
| 24 | Objectivity of treatment information | Use of professional photographs | 2 | 2 | 2 | 2 |
| 24 | Objectivity of treatment information | Use of patient photographs | 4 | 4 | 4 | 4 |
| 24 | Substantiation of claims | Materials or design | 3 | 3 | 3 | 3 |
| 24 | Substantiation of claims | Treatment process | 2 | 2 | 2 | 2 |
| 24 | Substantiation of claims | Outcome | 2 | 2 | 2 | 2 |
| 24 | Substantiation of claims | Superiority to comparators | 4 | 4 | 4 | 4 |
| 25 | Comprehensiveness of treatment information | Aim of treatment | 1 | 1 | 1 | 1 |
| 25 | Comprehensiveness of treatment information | Mode of action | 1 | 1 | 1 | 1 |
| 25 | Comprehensiveness of treatment information | Scope of treatment | 2 | 1 | 1 | 1 |
| 25 | Comprehensiveness of treatment information | Contraindications | 3 | 3 | 2 | 3 |
| 25 | Comprehensiveness of treatment information | Alternative treatments | 3 | 3 | 3 | 3 |
| 25 | Comprehensiveness of treatment information | Requirements for success | 2 | 3 | 3 | 3 |
| 25 | Comprehensiveness of treatment information | Likely treatment time | 1 | 1 | 1 | 1 |
| 25 | Comprehensiveness of treatment information | Need for long-term retention | 3 | 3 | 2 | 3 |
| 25 | Comprehensiveness of treatment information | Common risks of treatment | 2 | 2 | 2 | 2 |
| 25 | Comprehensiveness of treatment information | Common side effects of treatment | 3 | 3 | 3 | 3 |
| 25 | Presentation of treatment information | Accessibility of information | 1 | 1 | 1 | 1 |
| 25 | Presentation of treatment information | Consistency of information | 4 | 4 | 4 | 4 |
| 25 | Presentation of treatment information | Clarity of information | 3 | 3 | 3 | 3 |
| 25 | Objectivity of treatment information | Use of objective information | 3 | 3 | 3 | 3 |
| 25 | Objectivity of treatment information | Use of professional photographs | 3 | 3 | 3 | 3 |
| 25 | Objectivity of treatment information | Use of patient photographs | 4 | 4 | 4 | 4 |
| 25 | Substantiation of claims | Materials or design | 3 | 3 | 3 | 3 |
| 25 | Substantiation of claims | Treatment process | 4 | 4 | 4 | 4 |
| 25 | Substantiation of claims | Outcome | 3 | 3 | 3 | 3 |
| 25 | Substantiation of claims | Superiority to comparators | 3 | 3 | 3 | 3 |
| 26 | Comprehensiveness of treatment information | Aim of treatment | 1 | 2 | 1 | 1 |
| 26 | Comprehensiveness of treatment information | Mode of action | 2 | 3 | 3 | 3 |
| 26 | Comprehensiveness of treatment information | Scope of treatment | 3 | 3 | 3 | 3 |
| 26 | Comprehensiveness of treatment information | Contraindications | 2 | 3 | 3 | 3 |
| 26 | Comprehensiveness of treatment information | Alternative treatments | 3 | 3 | 3 | 3 |
| 26 | Comprehensiveness of treatment information | Requirements for success | 3 | 3 | 3 | 3 |
| 26 | Comprehensiveness of treatment information | Likely treatment time | 1 | 1 | 1 | 1 |
| 26 | Comprehensiveness of treatment information | Need for long-term retention | 3 | 3 | 3 | 3 |
| 26 | Comprehensiveness of treatment information | Common risks of treatment | 3 | 3 | 3 | 3 |
| 26 | Comprehensiveness of treatment information | Common side effects of treatment | 3 | 3 | 3 | 3 |
| 26 | Presentation of treatment information | Accessibility of information | 3 | 3 | 3 | 3 |
| 26 | Presentation of treatment information | Consistency of information | 2 | 3 | 3 | 3 |
| 26 | Presentation of treatment information | Clarity of information | 3 | 3 | 3 | 3 |
| 26 | Objectivity of treatment information | Use of objective information | 1 | 1 | 1 | 1 |
| 26 | Objectivity of treatment information | Use of professional photographs | 4 | 4 | 4 | 4 |
| 26 | Objectivity of treatment information | Use of patient photographs | 4 | 4 | 4 | 4 |
| 26 | Substantiation of claims | Materials or design | 2 | 2 | 2 | 2 |
| 26 | Substantiation of claims | Treatment process | 4 | 4 | 4 | 4 |
| 26 | Substantiation of claims | Outcome | 4 | 4 | 4 | 4 |
| 26 | Substantiation of claims | Superiority to comparators | 2 | 2 | 2 | 2 |
| 27 | Comprehensiveness of treatment information | Aim of treatment | 1 | 1 | 1 | 1 |
| 27 | Comprehensiveness of treatment information | Mode of action | 1 | 1 | 1 | 1 |
| 27 | Comprehensiveness of treatment information | Scope of treatment | 1 | 1 | 1 | 1 |
| 27 | Comprehensiveness of treatment information | Contraindications | 1 | 1 | 1 | 1 |
| 27 | Comprehensiveness of treatment information | Alternative treatments | 2 | 2 | 2 | 2 |
| 27 | Comprehensiveness of treatment information | Requirements for success | 1 | 1 | 1 | 1 |
| 27 | Comprehensiveness of treatment information | Likely treatment time | 1 | 1 | 1 | 1 |
| 27 | Comprehensiveness of treatment information | Need for long-term retention | 1 | 1 | 1 | 1 |
| 27 | Comprehensiveness of treatment information | Common risks of treatment | 3 | 3 | 3 | 3 |
| 27 | Comprehensiveness of treatment information | Common side effects of treatment | 3 | 3 | 3 | 3 |
| 27 | Presentation of treatment information | Accessibility of information | 1 | 1 | 1 | 1 |
| 27 | Presentation of treatment information | Consistency of information | 1 | 1 | 1 | 1 |
| 27 | Presentation of treatment information | Clarity of information | 3 | 3 | 3 | 3 |
| 27 | Objectivity of treatment information | Use of objective information | 1 | 1 | 1 | 1 |
| 27 | Objectivity of treatment information | Use of professional photographs | 4 | 4 | 4 | 4 |
| 27 | Objectivity of treatment information | Use of patient photographs | 4 | 4 | 4 | 4 |
| 27 | Substantiation of claims | Materials or design | 2 | 2 | 2 | 2 |
| 27 | Substantiation of claims | Treatment process | 2 | 2 | 2 | 2 |
| 27 | Substantiation of claims | Outcome | 3 | 3 | 3 | 3 |
| 27 | Substantiation of claims | Superiority to comparators | 3 | 3 | 3 | 3 |
| 28 | Comprehensiveness of treatment information | Aim of treatment | 1 | 1 | 1 | 1 |
| 28 | Comprehensiveness of treatment information | Mode of action | 3 | 2 | 3 | 3 |
| 28 | Comprehensiveness of treatment information | Scope of treatment | 3 | 3 | 3 | 3 |
| 28 | Comprehensiveness of treatment information | Contraindications | 3 | 3 | 3 | 3 |
| 28 | Comprehensiveness of treatment information | Alternative treatments | 3 | 3 | 3 | 3 |
| 28 | Comprehensiveness of treatment information | Requirements for success | 2 | 3 | 3 | 3 |
| 28 | Comprehensiveness of treatment information | Likely treatment time | 3 | 3 | 3 | 3 |
| 28 | Comprehensiveness of treatment information | Need for long-term retention | 2 | 3 | 3 | 3 |
| 28 | Comprehensiveness of treatment information | Common risks of treatment | 3 | 3 | 3 | 3 |
| 28 | Comprehensiveness of treatment information | Common side effects of treatment | 3 | 3 | 3 | 3 |
| 28 | Presentation of treatment information | Accessibility of information | 2 | 1 | 1 | 1 |
| 28 | Presentation of treatment information | Consistency of information | 4 | 4 | 4 | 4 |
| 28 | Presentation of treatment information | Clarity of information | 3 | 3 | 3 | 3 |
| 28 | Objectivity of treatment information | Use of objective information | 3 | 3 | 3 | 3 |
| 28 | Objectivity of treatment information | Use of professional photographs | 4 | 4 | 4 | 4 |
| 28 | Objectivity of treatment information | Use of patient photographs | 4 | 4 | 4 | 4 |
| 28 | Substantiation of claims | Materials or design | 3 | 3 | 3 | 3 |
| 28 | Substantiation of claims | Treatment process | 3 | 2 | 2 | 2 |
| 28 | Substantiation of claims | Outcome | 4 | 4 | 4 | 4 |
| 28 | Substantiation of claims | Superiority to comparators | 3 | 3 | 3 | 3 |
| 29 | Comprehensiveness of treatment information | Aim of treatment | 1 | 1 | 1 | 1 |
| 29 | Comprehensiveness of treatment information | Mode of action | 1 | 1 | 1 | 1 |
| 29 | Comprehensiveness of treatment information | Scope of treatment | 1 | 2 | 1 | 1 |
| 29 | Comprehensiveness of treatment information | Contraindications | 1 | 1 | 1 | 1 |
| 29 | Comprehensiveness of treatment information | Alternative treatments | 2 | 2 | 2 | 2 |
| 29 | Comprehensiveness of treatment information | Requirements for success | 1 | 2 | 1 | 1 |
| 29 | Comprehensiveness of treatment information | Likely treatment time | 1 | 1 | 1 | 1 |
| 29 | Comprehensiveness of treatment information | Need for long-term retention | 2 | 2 | 2 | 2 |
| 29 | Comprehensiveness of treatment information | Common risks of treatment | 3 | 3 | 3 | 3 |
| 29 | Comprehensiveness of treatment information | Common side effects of treatment | 2 | 2 | 2 | 2 |
| 29 | Presentation of treatment information | Accessibility of information | 1 | 1 | 1 | 1 |
| 29 | Presentation of treatment information | Consistency of information | 1 | 1 | 1 | 1 |
| 29 | Presentation of treatment information | Clarity of information | 3 | 3 | 3 | 3 |
| 29 | Objectivity of treatment information | Use of objective information | 1 | 1 | 1 | 1 |
| 29 | Objectivity of treatment information | Use of professional photographs | 4 | 4 | 4 | 4 |
| 29 | Objectivity of treatment information | Use of patient photographs | 3 | 3 | 3 | 3 |
| 29 | Substantiation of claims | Materials or design | 2 | 2 | 2 | 2 |
| 29 | Substantiation of claims | Treatment process | 4 | 4 | 4 | 4 |
| 29 | Substantiation of claims | Outcome | 4 | 4 | 4 | 4 |
| 29 | Substantiation of claims | Superiority to comparators | 4 | 4 | 4 | 4 |
| 30 | Comprehensiveness of treatment information | Aim of treatment | 1 | 1 | 1 | 1 |
| 30 | Comprehensiveness of treatment information | Mode of action | 1 | 1 | 1 | 1 |
| 30 | Comprehensiveness of treatment information | Scope of treatment | 1 | 1 | 1 | 1 |
| 30 | Comprehensiveness of treatment information | Contraindications | 3 | 3 | 3 | 3 |
| 30 | Comprehensiveness of treatment information | Alternative treatments | 3 | 3 | 3 | 3 |
| 30 | Comprehensiveness of treatment information | Requirements for success | 3 | 3 | 3 | 3 |
| 30 | Comprehensiveness of treatment information | Likely treatment time | 1 | 1 | 1 | 1 |
| 30 | Comprehensiveness of treatment information | Need for long-term retention | 3 | 3 | 3 | 3 |
| 30 | Comprehensiveness of treatment information | Common risks of treatment | 3 | 3 | 3 | 3 |
| 30 | Comprehensiveness of treatment information | Common side effects of treatment | 3 | 3 | 3 | 3 |
| 30 | Presentation of treatment information | Accessibility of information | 1 | 1 | 1 | 1 |
| 30 | Presentation of treatment information | Consistency of information | 1 | 1 | 1 | 1 |
| 30 | Presentation of treatment information | Clarity of information | 3 | 3 | 3 | 3 |
| 30 | Objectivity of treatment information | Use of objective information | 1 | 1 | 1 | 1 |
| 30 | Objectivity of treatment information | Use of professional photographs | 1 | 1 | 1 | 1 |
| 30 | Objectivity of treatment information | Use of patient photographs | 4 | 4 | 4 | 4 |
| 30 | Substantiation of claims | Materials or design | 4 | 4 | 4 | 4 |
| 30 | Substantiation of claims | Treatment process | 2 | 2 | 2 | 2 |
| 30 | Substantiation of claims | Outcome | 2 | 2 | 2 | 2 |
| 30 | Substantiation of claims | Superiority to comparators | 2 | 2 | 2 | 2 |
| 31 | Comprehensiveness of treatment information | Aim of treatment | 1 | 1 | 1 | 1 |
| 31 | Comprehensiveness of treatment information | Mode of action | 1 | 1 | 1 | 1 |
| 31 | Comprehensiveness of treatment information | Scope of treatment | 1 | 1 | 1 | 1 |
| 31 | Comprehensiveness of treatment information | Contraindications | 2 | 3 | 3 | 3 |
| 31 | Comprehensiveness of treatment information | Alternative treatments | 3 | 3 | 3 | 3 |
| 31 | Comprehensiveness of treatment information | Requirements for success | 1 | 1 | 1 | 1 |
| 31 | Comprehensiveness of treatment information | Likely treatment time | 1 | 1 | 1 | 1 |
| 31 | Comprehensiveness of treatment information | Need for long-term retention | 1 | 1 | 1 | 1 |
| 31 | Comprehensiveness of treatment information | Common risks of treatment | 3 | 3 | 3 | 3 |
| 31 | Comprehensiveness of treatment information | Common side effects of treatment | 1 | 2 | 1 | 1 |
| 31 | Presentation of treatment information | Accessibility of information | 1 | 1 | 2 | 1 |
| 31 | Presentation of treatment information | Consistency of information | 1 | 1 | 1 | 1 |
| 31 | Presentation of treatment information | Clarity of information | 3 | 3 | 3 | 3 |
| 31 | Objectivity of treatment information | Use of objective information | 1 | 1 | 1 | 1 |
| 31 | Objectivity of treatment information | Use of professional photographs | 1 | 1 | 1 | 1 |
| 31 | Objectivity of treatment information | Use of patient photographs | 4 | 4 | 4 | 4 |
| 31 | Substantiation of claims | Materials or design | 2 | 3 | 2 | 2 |
| 31 | Substantiation of claims | Treatment process | 1 | 2 | 2 | 2 |
| 31 | Substantiation of claims | Outcome | 2 | 2 | 2 | 2 |
| 31 | Substantiation of claims | Superiority to comparators | 2 | 2 | 2 | 2 |
| 32 | Comprehensiveness of treatment information | Aim of treatment | 1 | 1 | 1 | 1 |
| 32 | Comprehensiveness of treatment information | Mode of action | 1 | 1 | 1 | 1 |
| 32 | Comprehensiveness of treatment information | Scope of treatment | 1 | 1 | 1 | 1 |
| 32 | Comprehensiveness of treatment information | Contraindications | 3 | 3 | 3 | 3 |
| 32 | Comprehensiveness of treatment information | Alternative treatments | 3 | 3 | 3 | 3 |
| 32 | Comprehensiveness of treatment information | Requirements for success | 2 | 2 | 2 | 2 |
| 32 | Comprehensiveness of treatment information | Likely treatment time | 1 | 1 | 1 | 1 |
| 32 | Comprehensiveness of treatment information | Need for long-term retention | 1 | 1 | 1 | 1 |
| 32 | Comprehensiveness of treatment information | Common risks of treatment | 3 | 3 | 3 | 3 |
| 32 | Comprehensiveness of treatment information | Common side effects of treatment | 1 | 1 | 1 | 1 |
| 32 | Presentation of treatment information | Accessibility of information | 1 | 1 | 1 | 1 |
| 32 | Presentation of treatment information | Consistency of information | 3 | 3 | 3 | 3 |
| 32 | Presentation of treatment information | Clarity of information | 1 | 1 | 1 | 1 |
| 32 | Objectivity of treatment information | Use of objective information | 1 | 1 | 1 | 1 |
| 32 | Objectivity of treatment information | Use of professional photographs | 3 | 3 | 3 | 3 |
| 32 | Objectivity of treatment information | Use of patient photographs | 3 | 3 | 3 | 3 |
| 32 | Substantiation of claims | Materials or design | 3 | 3 | 3 | 3 |
| 32 | Substantiation of claims | Treatment process | 2 | 2 | 2 | 2 |
| 32 | Substantiation of claims | Outcome | 2 | 2 | 2 | 2 |
| 32 | Substantiation of claims | Superiority to comparators | 2 | 2 | 2 | 2 |
| 33 | Comprehensiveness of treatment information | Aim of treatment | 1 | 1 | 1 | 1 |
| 33 | Comprehensiveness of treatment information | Mode of action | 3 | 3 | 3 | 3 |
| 33 | Comprehensiveness of treatment information | Scope of treatment | 3 | 3 | 3 | 3 |
| 33 | Comprehensiveness of treatment information | Contraindications | 2 | 3 | 3 | 3 |
| 33 | Comprehensiveness of treatment information | Alternative treatments | 3 | 3 | 3 | 3 |
| 33 | Comprehensiveness of treatment information | Requirements for success | 1 | 1 | 1 | 1 |
| 33 | Comprehensiveness of treatment information | Likely treatment time | 1 | 1 | 1 | 1 |
| 33 | Comprehensiveness of treatment information | Need for long-term retention | 3 | 3 | 3 | 3 |
| 33 | Comprehensiveness of treatment information | Common risks of treatment | 3 | 3 | 3 | 3 |
| 33 | Comprehensiveness of treatment information | Common side effects of treatment | 1 | 2 | 1 | 1 |
| 33 | Presentation of treatment information | Accessibility of information | 2 | 1 | 1 | 1 |
| 33 | Presentation of treatment information | Consistency of information | 1 | 1 | 1 | 1 |
| 33 | Presentation of treatment information | Clarity of information | 3 | 3 | 3 | 3 |
| 33 | Objectivity of treatment information | Use of objective information | 1 | 1 | 1 | 1 |
| 33 | Objectivity of treatment information | Use of professional photographs | 4 | 4 | 4 | 4 |
| 33 | Objectivity of treatment information | Use of patient photographs | 3 | 3 | 3 | 3 |
| 33 | Substantiation of claims | Materials or design | 2 | 2 | 2 | 3 |
| 33 | Substantiation of claims | Treatment process | 1 | 2 | 2 | 2 |
| 33 | Substantiation of claims | Outcome | 2 | 2 | 2 | 2 |
| 33 | Substantiation of claims | Superiority to comparators | 3 | 3 | 3 | 3 |
| 34 | Comprehensiveness of treatment information | Aim of treatment | 1 | 1 | 1 | 1 |
| 34 | Comprehensiveness of treatment information | Mode of action | 1 | 1 | 1 | 1 |
| 34 | Comprehensiveness of treatment information | Scope of treatment | 1 | 1 | 1 | 1 |
| 34 | Comprehensiveness of treatment information | Contraindications | 3 | 3 | 3 | 3 |
| 34 | Comprehensiveness of treatment information | Alternative treatments | 3 | 3 | 3 | 3 |
| 34 | Comprehensiveness of treatment information | Requirements for success | 2 | 2 | 2 | 2 |
| 34 | Comprehensiveness of treatment information | Likely treatment time | 1 | 1 | 1 | 1 |
| 34 | Comprehensiveness of treatment information | Need for long-term retention | 1 | 1 | 1 | 1 |
| 34 | Comprehensiveness of treatment information | Common risks of treatment | 3 | 3 | 3 | 3 |
| 34 | Comprehensiveness of treatment information | Common side effects of treatment | 2 | 2 | 2 | 2 |
| 34 | Presentation of treatment information | Accessibility of information | 1 | 1 | 1 | 1 |
| 34 | Presentation of treatment information | Consistency of information | 1 | 1 | 1 | 1 |
| 34 | Presentation of treatment information | Clarity of information | 3 | 3 | 3 | 3 |
| 34 | Objectivity of treatment information | Use of objective information | 1 | 1 | 1 | 1 |
| 34 | Objectivity of treatment information | Use of professional photographs | 4 | 4 | 4 | 4 |
| 34 | Objectivity of treatment information | Use of patient photographs | 4 | 4 | 4 | 4 |
| 34 | Substantiation of claims | Materials or design | 4 | 4 | 4 | 4 |
| 34 | Substantiation of claims | Treatment process | 2 | 2 | 2 | 2 |
| 34 | Substantiation of claims | Outcome | 2 | 2 | 2 | 2 |
| 34 | Substantiation of claims | Superiority to comparators | 3 | 3 | 3 | 3 |
| 35 | Comprehensiveness of treatment information | Aim of treatment | 1 | 1 | 1 | 1 |
| 35 | Comprehensiveness of treatment information | Mode of action | 1 | 1 | 1 | 1 |
| 35 | Comprehensiveness of treatment information | Scope of treatment | 1 | 1 | 1 | 1 |
| 35 | Comprehensiveness of treatment information | Contraindications | 2 | 2 | 2 | 2 |
| 35 | Comprehensiveness of treatment information | Alternative treatments | 3 | 3 | 3 | 3 |
| 35 | Comprehensiveness of treatment information | Requirements for success | 2 | 2 | 2 | 2 |
| 35 | Comprehensiveness of treatment information | Likely treatment time | 1 | 1 | 1 | 1 |
| 35 | Comprehensiveness of treatment information | Need for long-term retention | 1 | 1 | 1 | 1 |
| 35 | Comprehensiveness of treatment information | Common risks of treatment | 3 | 3 | 3 | 3 |
| 35 | Comprehensiveness of treatment information | Common side effects of treatment | 3 | 3 | 3 | 3 |
| 35 | Presentation of treatment information | Accessibility of information | 1 | 1 | 1 | 1 |
| 35 | Presentation of treatment information | Consistency of information | 3 | 3 | 3 | 3 |
| 35 | Presentation of treatment information | Clarity of information | 3 | 3 | 3 | 3 |
| 35 | Objectivity of treatment information | Use of objective information | 1 | 1 | 1 | 1 |
| 35 | Objectivity of treatment information | Use of professional photographs | 3 | 3 | 3 | 3 |
| 35 | Objectivity of treatment information | Use of patient photographs | 3 | 3 | 3 | 3 |
| 35 | Substantiation of claims | Materials or design | 3 | 3 | 3 | 3 |
| 35 | Substantiation of claims | Treatment process | 2 | 2 | 2 | 2 |
| 35 | Substantiation of claims | Outcome | 2 | 2 | 2 | 2 |
| 35 | Substantiation of claims | Superiority to comparators | 3 | 3 | 3 | 3 |
| 36 | Comprehensiveness of treatment information | Aim of treatment | 3 | 3 | 3 | 3 |
| 36 | Comprehensiveness of treatment information | Mode of action | 3 | 3 | 3 | 3 |
| 36 | Comprehensiveness of treatment information | Scope of treatment | 3 | 3 | 3 | 3 |
| 36 | Comprehensiveness of treatment information | Contraindications | 3 | 3 | 3 | 3 |
| 36 | Comprehensiveness of treatment information | Alternative treatments | 3 | 3 | 3 | 3 |
| 36 | Comprehensiveness of treatment information | Requirements for success | 2 | 2 | 2 | 2 |
| 36 | Comprehensiveness of treatment information | Likely treatment time | 3 | 3 | 3 | 3 |
| 36 | Comprehensiveness of treatment information | Need for long-term retention | 2 | 2 | 2 | 2 |
| 36 | Comprehensiveness of treatment information | Common risks of treatment | 3 | 3 | 3 | 3 |
| 36 | Comprehensiveness of treatment information | Common side effects of treatment | 2 | 2 | 2 | 2 |
| 36 | Presentation of treatment information | Accessibility of information | 1 | 1 | 1 | 1 |
| 36 | Presentation of treatment information | Consistency of information | 4 | 4 | 4 | 4 |
| 36 | Presentation of treatment information | Clarity of information | 3 | 3 | 3 | 3 |
| 36 | Objectivity of treatment information | Use of objective information | 1 | 1 | 1 | 1 |
| 36 | Objectivity of treatment information | Use of professional photographs | 3 | 3 | 3 | 3 |
| 36 | Objectivity of treatment information | Use of patient photographs | 3 | 3 | 3 | 3 |
| 36 | Substantiation of claims | Materials or design | 3 | 3 | 3 | 3 |
| 36 | Substantiation of claims | Treatment process | 2 | 2 | 2 | 2 |
| 36 | Substantiation of claims | Outcome | 3 | 3 | 3 | 3 |
| 36 | Substantiation of claims | Superiority to comparators | 3 | 3 | 3 | 3 |
| 37 | Comprehensiveness of treatment information | Aim of treatment | 1 | 1 | 1 | 1 |
| 37 | Comprehensiveness of treatment information | Mode of action | 1 | 1 | 1 | 1 |
| 37 | Comprehensiveness of treatment information | Scope of treatment | 1 | 1 | 1 | 1 |
| 37 | Comprehensiveness of treatment information | Contraindications | 2 | 2 | 2 | 2 |
| 37 | Comprehensiveness of treatment information | Alternative treatments | 3 | 2 | 2 | 2 |
| 37 | Comprehensiveness of treatment information | Requirements for success | 1 | 1 | 1 | 1 |
| 37 | Comprehensiveness of treatment information | Likely treatment time | 1 | 1 | 1 | 1 |
| 37 | Comprehensiveness of treatment information | Need for long-term retention | 2 | 1 | 1 | 1 |
| 37 | Comprehensiveness of treatment information | Common risks of treatment | 3 | 3 | 3 | 3 |
| 37 | Comprehensiveness of treatment information | Common side effects of treatment | 1 | 1 | 1 | 1 |
| 37 | Presentation of treatment information | Accessibility of information | 3 | 3 | 3 | 3 |
| 37 | Presentation of treatment information | Consistency of information | 2 | 3 | 3 | 3 |
| 37 | Presentation of treatment information | Clarity of information | 3 | 3 | 3 | 3 |
| 37 | Objectivity of treatment information | Use of objective information | 1 | 1 | 1 | 1 |
| 37 | Objectivity of treatment information | Use of professional photographs | 4 | 4 | 4 | 4 |
| 37 | Objectivity of treatment information | Use of patient photographs | 3 | 3 | 3 | 3 |
| 37 | Substantiation of claims | Materials or design | 3 | 3 | 3 | 3 |
| 37 | Substantiation of claims | Treatment process | 2 | 2 | 2 | 2 |
| 37 | Substantiation of claims | Outcome | 2 | 2 | 2 | 2 |
| 37 | Substantiation of claims | Superiority to comparators | 2 | 2 | 2 | 3 |
| 38 | Comprehensiveness of treatment information | Aim of treatment | 1 | 1 | 1 | 1 |
| 38 | Comprehensiveness of treatment information | Mode of action | 1 | 1 | 1 | 1 |
| 38 | Comprehensiveness of treatment information | Scope of treatment | 1 | 1 | 1 | 1 |
| 38 | Comprehensiveness of treatment information | Contraindications | 1 | 2 | 2 | 2 |
| 38 | Comprehensiveness of treatment information | Alternative treatments | 3 | 3 | 3 | 3 |
| 38 | Comprehensiveness of treatment information | Requirements for success | 2 | 3 | 2 | 2 |
| 38 | Comprehensiveness of treatment information | Likely treatment time | 1 | 1 | 1 | 1 |
| 38 | Comprehensiveness of treatment information | Need for long-term retention | 1 | 1 | 1 | 1 |
| 38 | Comprehensiveness of treatment information | Common risks of treatment | 3 | 3 | 3 | 3 |
| 38 | Comprehensiveness of treatment information | Common side effects of treatment | 3 | 3 | 3 | 3 |
| 38 | Presentation of treatment information | Accessibility of information | 3 | 2 | 3 | 3 |
| 38 | Presentation of treatment information | Consistency of information | 1 | 1 | 1 | 1 |
| 38 | Presentation of treatment information | Clarity of information | 3 | 3 | 3 | 3 |
| 38 | Objectivity of treatment information | Use of objective information | 1 | 1 | 1 | 1 |
| 38 | Objectivity of treatment information | Use of professional photographs | 4 | 4 | 4 | 4 |
| 38 | Objectivity of treatment information | Use of patient photographs | 3 | 3 | 3 | 3 |
| 38 | Substantiation of claims | Materials or design | 2 | 3 | 2 | 2 |
| 38 | Substantiation of claims | Treatment process | 2 | 2 | 2 | 2 |
| 38 | Substantiation of claims | Outcome | 4 | 4 | 4 | 4 |
| 38 | Substantiation of claims | Superiority to comparators | 3 | 3 | 3 | 3 |
| 39 | Comprehensiveness of treatment information | Aim of treatment | 3 | 3 | 3 | 3 |
| 39 | Comprehensiveness of treatment information | Mode of action | 3 | 3 | 3 | 3 |
| 39 | Comprehensiveness of treatment information | Scope of treatment | 2 | 2 | 2 | 2 |
| 39 | Comprehensiveness of treatment information | Contraindications | 2 | 2 | 2 | 2 |
| 39 | Comprehensiveness of treatment information | Alternative treatments | 3 | 3 | 3 | 3 |
| 39 | Comprehensiveness of treatment information | Requirements for success | 3 | 3 | 3 | 3 |
| 39 | Comprehensiveness of treatment information | Likely treatment time | 3 | 3 | 3 | 3 |
| 39 | Comprehensiveness of treatment information | Need for long-term retention | 3 | 3 | 3 | 3 |
| 39 | Comprehensiveness of treatment information | Common risks of treatment | 3 | 3 | 3 | 3 |
| 39 | Comprehensiveness of treatment information | Common side effects of treatment | 3 | 3 | 3 | 3 |
| 39 | Presentation of treatment information | Accessibility of information | 1 | 1 | 1 | 1 |
| 39 | Presentation of treatment information | Consistency of information | 4 | 4 | 4 | 4 |
| 39 | Presentation of treatment information | Clarity of information | 1 | 1 | 1 | 1 |
| 39 | Objectivity of treatment information | Use of objective information | 1 | 1 | 1 | 1 |
| 39 | Objectivity of treatment information | Use of professional photographs | 3 | 3 | 3 | 3 |
| 39 | Objectivity of treatment information | Use of patient photographs | 4 | 4 | 4 | 4 |
| 39 | Substantiation of claims | Materials or design | 4 | 4 | 4 | 4 |
| 39 | Substantiation of claims | Treatment process | 4 | 4 | 4 | 4 |
| 39 | Substantiation of claims | Outcome | 4 | 4 | 4 | 4 |
| 39 | Substantiation of claims | Superiority to comparators | 3 | 3 | 3 | 3 |

Score

1 = Complete information/Yes/Claims are supported by appropriate evidence

2 = Partial information/Partial/Claims are reasonable but not supported by evidence

3 = No information/No/Claims are exaggerated or use inappropriate citation

4 = No FAQ/Not used/No claims are made
